# Plasmonically Enhanced Reflectance of Heat Radiation from Low-Bandgap Semiconductor Microinclusions

**DOI:** 10.1038/s41598-017-05630-4

**Published:** 2017-07-18

**Authors:** Janika Tang, Vaibhav Thakore, Tapio Ala-Nissila

**Affiliations:** 1 COMP CoE at the Department of Applied Physics, Aalto University School of Science, FIN-00076 Aalto, Espoo Finland; 2 Department of Physics, Brown University, Providence, Rhode Island 02912-1843 USA; 3 Department of Mathematical Sciences and Department of Physics, Loughborough University, Loughborough, LE11 3TU UK

## Abstract

Increased reflectance from the inclusion of highly scattering particles at low volume fractions in an insulating dielectric offers a promising way to reduce radiative thermal losses at high temperatures. Here, we investigate plasmonic resonance driven enhanced scattering from microinclusions of low-bandgap semiconductors (InP, Si, Ge, PbS, InAs and Te) in an insulating composite to tailor its infrared reflectance for minimizing thermal losses from radiative transfer. To this end, we compute the spectral properties of the microcomposites using Monte Carlo modeling and compare them with results from Fresnel equations. The role of particle size-dependent Mie scattering and absorption efficiencies, and, scattering anisotropy are studied to identify the optimal microinclusion size and material parameters for maximizing the reflectance of the thermal radiation. For composites with Si and Ge microinclusions we obtain reflectance efficiencies of 57–65% for the incident blackbody radiation from sources at temperatures in the range 400–1600 °C. Furthermore, we observe a broadbanding of the reflectance spectra from the plasmonic resonances due to charge carriers generated from defect states within the semiconductor bandgap. Our results thus open up the possibility of developing efficient high-temperature thermal insulators through use of the low-bandgap semiconductor microinclusions in insulating dielectrics.

## Introduction

Efficient thermal insulation at a given temperature must reduce unwanted heat exchange with the surrounding environment that occurs primarily through the twin modes of conductive and radiative heat transfer. Designing an efficient thermal insulator thus involves a subtle tradeoff between minimizing conductive heat loss by optimizing the porosity of an insulating material, *e*.*g*. with microstructured air-pockets, and simultaneously ensuring that there is no significant thermal loss through increased radiative heat transfer^1–5^. This approach works well for low temperature applications. However, under high temperature conditions radiative heat transfer becomes the dominant mode of thermal losses^2^. In such cases, decreasing the porosity of the material to prevent radiative losses becomes unfeasible as an alternative because it inevitably also leads to higher conductive losses. Therefore, a strategy for designing an efficient thermal insulator for high temperature applications must carefully balance the two phenomena. The ability to tailor the broadband infrared reflectance to minimize radiative losses has important implications for providing efficient thermal insulation under high temperature conditions and in applications such as furnaces, fire protection, gas-turbine engines, redirecting heat in photovoltaic systems, in energy-efficient buildings, etc.^6–9^.

A vast amount of literature exists on new materials for coatings and paints doped with metal/metal-oxide pigments or dyes that is focused on obtaining increased absorbance or reflectance of solar radiation^10–13^. These coatings or paints are referred to as ‘cool’ or ‘hot’ depending on whether they enhance diffuse reflectance through scattering or enable spectrally selective absorption in the near-infrared wavelength (NIR) regime^14–18^. These materials, while excellent for facilitating effective harnessing of solar energy in photovoltaic devices or for thermal management in buildings and vehicles, are however not suitable for use as thermal insulators at high temperatures because of their high thermal conductivities^11, 19^. Multilayer dielectric materials used in thermal barrier coatings offer an alternative but are prohibitively expensive to fabricate and maintain for structurally complex systems^20, 21^. In this regard, an attractive low-cost alternative is offered by thermal insulators such as aerogels that are characterized by remarkably low thermal conductivities. However, aerogels are almost transparent to the NIR wavelengths (3–8 μm) rendering them unsuitable for use in high temperature environments^2^. Aerogel based thermal insulators therefore require the use of opacifiers for improving insulation at high temperatures wherein radiative transfer losses dominate^2^. Opacifiers are typically particles of refractory metal-oxides, carbides or nitrides that are randomly distributed at high mass fractions in aerogels to enable multiple scattering of thermal radiation and thereby improve diffuse reflectance^2, 3, 22, 23^.

Recently, localized surface plasmon resonances (LSPRs) in randomly distributed metallic nanoparticles on surfaces and in films have been exploited to demonstrate controlled reflectance^14, 19, 24^. LSPRs arise due to a confinement of the collective oscillations (plasmons) of free charge carriers on the surface of a micro or nanoparticle driven by the electromagnetic field of the incident radiation of wavelength greater than or comparable to the size of the particle^25^. These multipolar collective oscillations of charge carriers excited by the incident radiation absorb energy close to resonance and re-radiate it in all possible directions. This results in enhanced scattering and absorption resonances that can be controlled with the geometry, size, dielectric environment and the spatial distribution of the particles^14, 19, 25–30^. Although LSPRs in metallic particles can be tailored to modify reflectance, the tunability of their frequency response lies mostly in either the ultraviolet or visible spectrum of the electromagnetic radiation. Furthermore, besides the regime of frequency response, the high thermal conductivity of metallic particles makes them unsuitable for use as opacifiers in insulators for high temperature applications. However, low-bandgap semiconductors, characterized by relatively low-thermal conductivities, exhibit LSPRs that can be excited by the incident heat radiation in the infrared regime^30^. Based on the Drude model for charge transport, the characteristic plasma frequency *ω*_*p*_ of a material that determines its optical response close to resonance is directly proportional to the square root of its free carrier concentration *N i*.*e*. ${\omega }_{p}=\sqrt{N{e}^{2}/{m}^{\ast }{\varepsilon }_{o}}$ (where, *e* and *m* ^*^ are the charge and the effective mass of the charge carriers and *ε*_*o*_ is the permittivity of the free space). In contrast to metals, the free charge carrier concentration in semiconductors can be controlled precisely through doping. Thus, the use of low bandgap semiconductor inclusions as opacifiers for tailoring the optical spectra of the composites will allow for a continuous tunability of the LSPR frequencies. Low-bandgap semiconductor inclusions with an appropriate bandgap and carrier concentration therefore hold excellent promise as opacifiers in high temperature insulators. In this study, we focus our investigation on the effect of the plasmonic resonance induced enhanced scattering on the diffuse reflectance of thermal radiation from insulator dielectrics with low-bandgap semiconducting microinclusions.

Radiative heat transport in materials can be modeled using several different methods that include numerical methods for solving the radiative transfer equation^31^, ray-tracing based on geometrical optics^32–34^, flux based methods^35–38^ and Monte Carlo models^39–42^. Numerical methods for solving the radiative transfer equation that employ a finite number of angular intensities such as the discrete transfer method (DTM), discrete ordinates method (DOM) and the finite volume method (FVM) typically require some kind of an assumption of angular isotropy for scattering^31^. The radiation element method by the ray emission model (REM^2^) also employs a finite number of angular intensities but gets around this difficulty by considering scattering anisotropy through the use of a delta function approximation for the scattering phase function^43^. In general, these methods can be applied to complex geometries but they also tend to limit radiation transport to certain discrete directions thereby affecting their accuracy. The flux-based methods employ coupled ordinary differential equations to model radiative transport in two-dimensional media along the normal direction^35–38^. The two-flux Kubelka-Munk (KM)^35^ and the extended KM radiative transfer models^37^, frequently employed due to their ease of implementation, are some of the oldest flux-based methods available for diffuse and collimated incident radiation respectively. However, the KM methods are applicable only to optically thick films with non-absorbing particles or to films with highly scattering and weakly absorbing particles with size-parameters larger than the Rayleigh limit^37^. Improvements upon the KM models account for backward and forward fluxes of diffuse and collimated radiation separately through the incorporation of additional flux channels^37^. The most widely used of these methods is the generalized four-flux model due to Vargas and Niklasson^36, 37^ based on the four-flux model proposed by Maheu *et al*.^38^. However, in the case of media characterized by large anisotropic scattering the generalized four-flux method requires an evaluation of the average path-length parameters using the extended Hartels theory^44^. On the other hand, Monte Carlo methods based on tracking packets of incident radiation (henceforth referred to as photons) in two or three dimensions are highly accurate and applicable to anisotropic media with multiple scattering without requiring the evaluation of any average path-length parameters or the use of a finite number of angular intensities^45^. Thus, here we use a Monte Carlo method in conjunction with Mie theory for modeling radiation transport in a microcomposite dielectric insulator with spherical semiconducting microinclusions at low volume fractions.

Recently, Slovick *et al*. have experimentally demonstrated the tailoring of the diffuse infrared reflectance of up to 90% for LPC paints with microscale inclusions of single-crystal hexagonal Boron Nitride platelets (h-BN) albeit at an unusually high h-BN volume fraction of *f* = 0.5^11^. Gonome *et al*. have also demonstrated up to 90% near-infrared broadband reflectances for cool coatings with submicron copper-oxide (CuO) particles at low volume fractions ranging from *f* = 0.02 to 0.05^46^. However, these high reflectances were obtained for coatings on highly reflecting white substrates while coatings on black substrates yielded significantly lower reflectances of about 35–40%^46^. Also, currently there exist no studies that systematically investigate the effect of Mie parameters for microparticles on maximizing the reflectance of incident thermal radiation from composites or coatings. Thus, the key objective of our study is to understand the role of the particle size-dependent Mie scattering *Q*_sca_ and absorption *Q*_abs_ efficiencies and the scattering anisotropy *g* in designing insulating composites with low-bandgap semiconductor microinclusions at low volume fractions *f* to maximize the reflectance of the incident thermal radiation. To this end, we compute infrared spectra for insulating dielectric composites with semiconductor microparticle inclusions of indium arsenide (InAs), lead sulphide (PbS), indium phosphide (InP), silicon (Si), germanium (Ge) and tellurium (Te), with direct and indirect bandgaps ranging from 0.3 to 1.4 eV. We then identify the optimal particle size of inclusions required to obtain maximal reflectance by quantifying the total reflectance from the insulating microcomposites in terms of a reflectance efficiency parameter *η* for incident thermal radiation originating from black-body sources at various temperatures *T*_*s*_. Additionally, we examine the effect of scattering from the microparticles on diffuse reflectance by comparing results from the Monte Carlo modeling with those from Fresnel equations based on the effective medium theory (EMT). The Fresnel equations take into account interference effects arising from the partial reflectance of the incident thermal radiation at the composite-ambient interfaces but do not account for scattering from the inclusions.

## Results

**Figure 1:**
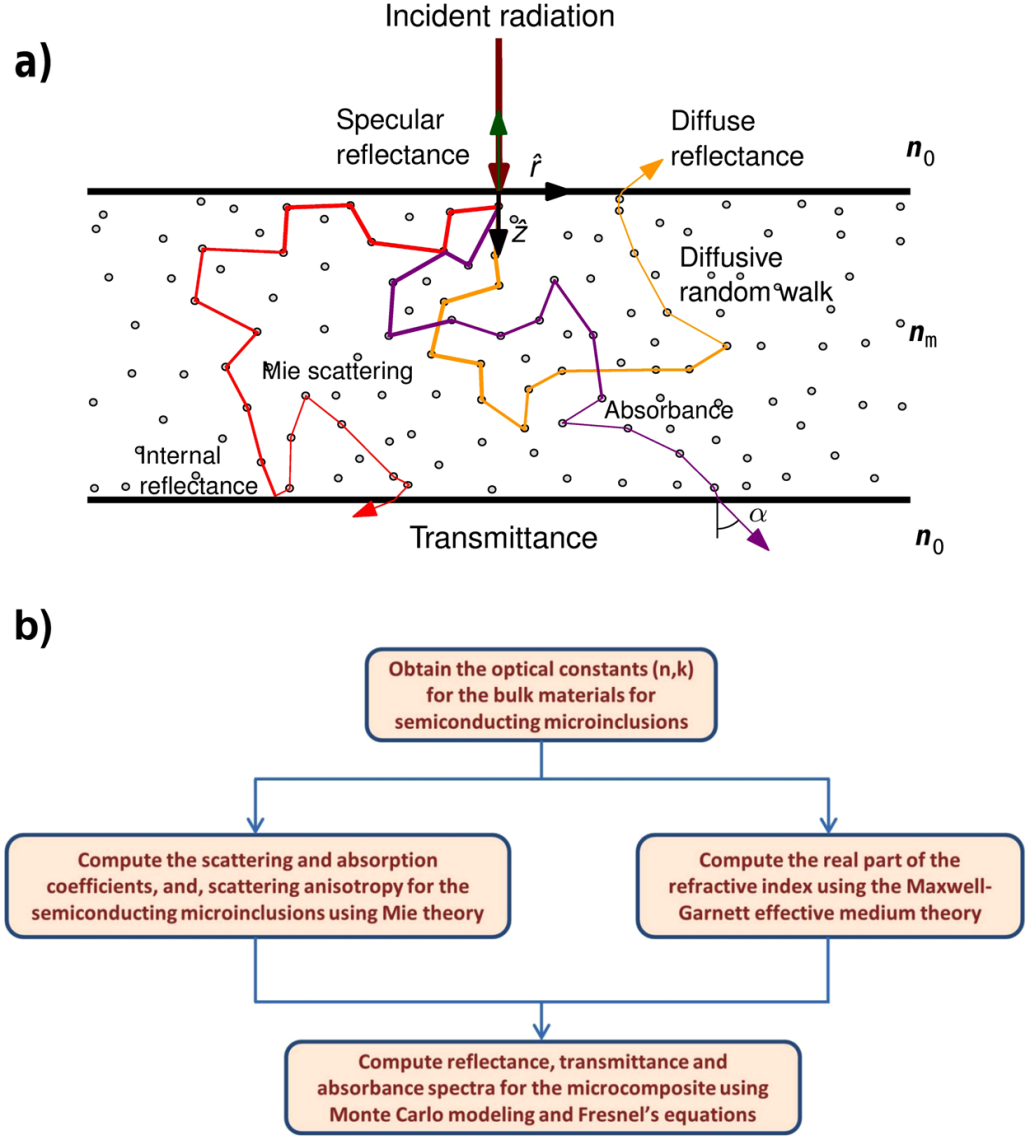
Computational methods employed to obtain the optical spectra for the insulator composites with semiconductor microinclusions. (**a**) Schematic illustrating the Monte Carlo model of propagating photons inside composites with scattering microinclusions for modeling the transport of the incident thermal radiation. An infinitesimally thin beam of incident photons is scattered within the microcomposite until either the photons are absorbed or they exit the system. The randomly distributed small open circles represent microinclusions that serve as scattering and absorption centers for the photons. The decrease in the thickness of the color trajectories in the schematic represents the decrements in the photon weights as they execute random motion in the microcomposite layer. The direction of photon exit from the composite, characterized by the angle *α* in the Monte Carlo model, varies with each random trajectory and for a large number of photons cumulatively gives rise to diffuse reflectance or transmittance. (**b**) Work-flow for the computation of the simulation parameters based on Mie theory and MG-EMT for use with the Monte Carlo method.

We first examine the results from the Mie theory calculations for the scattering (*Q*_sca_) and absorption (*Q*_abs_) efficiencies, and, the asymmetry factor *g* for Ge, Si, PbS, InP, InAs and Te microparticles of various diameters *d*. This is followed by results obtained from Monte Carlo modeling and Fresnel equations for the reflectance and absorbance spectra. Both the Monte Carlo model and the Fresnel equations employ the computed Mie parameters *Q*_sca_ and *Q*_abs_ (see Methods, Equations 3) as inputs for the computation of the optical spectra while the Monte Carlo model requires the additional use of the asymmetry factor *g* as well. For this study, we obtain the experimentally determined bulk values for the complex refractive indices of these materials from Palik^47^(See Supplementary Information (SI), SI Figure 2). For birefringent Te, the bulk refractive indices are averaged over the ordinary and the extraordinary directions. Arguably, our choice of the low-bandgap semiconductor materials for microinclusions is a *priori* somewhat arbitrary. However, it is designed to understand the scattering and reflectance properties of composites with microinclusions of materials characterized by a range of direct (PbS, InAs, InP, Te) and indirect bandgaps (Si, Ge), and, elemental and compound semiconductors that are already in widespread use or are easy to synthesize in bulk using the chemical route at low cost^48, 49^. Figure 1a,b describe the Monte Carlo model and the procedure employed in the computation of the optical spectra for the microcomposites, respectively. For further details, however, the reader is referred to the section on Theory and Methods following Conclusions.

### Mie scattering from semiconductor microinclusions

**Figure 2:**
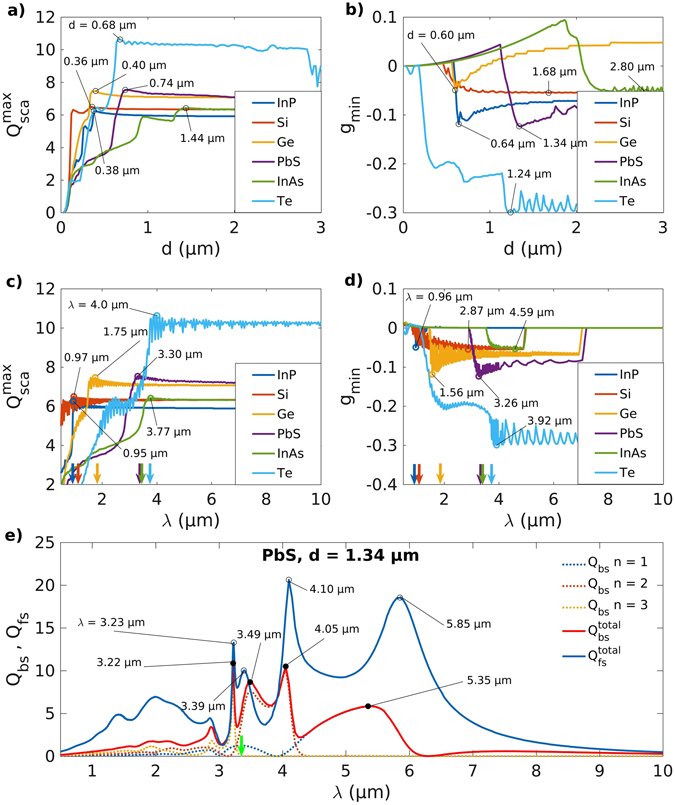
Optimal Mie scattering parameters for obtaining enhanced reflectance of incident heat radiation. (**a**,**c**) Maxima in scattering efficiency *Q*_sca_, and, (**b**,**d**) minima in anisotropy factor *g* as a function of the microinclusion size *d* and the wavelength *λ* for the different materials considered here. (**e**) Forward (*Q*_fs_) and back (*Q*_bs_) scattering efficiencies along with a deconvolution of *Q*_bs_ into contributions from the dipole and higher order modes (*n* = 1, 2, and 3) as a function of the wavelength *λ* for PbS microinclusions of diameter ${d}_{{g}_{{\rm{\min }}}}=1.34$ μm corresponding to the minima *g*_min_ in scattering anisotropy factor. The colored vertical arrows represent the bandgap wavelengths *λ*_bg_ and in (**e**) the green vertical arrow represents the *λ*_bg_ for PbS. A sharp switch from forward (+*g*) to backward scattering (−*g*) with an increase in the particle diameter *d* close to the bandgap wavelength (*λ*_bg_) in all materials points to the presence of Fano resonances^50, 51^. An inverse hierarchy of scattering resonances is also observed wherein the octupole (*n* = 3) mode is the strongest followed by quadrupole and dipole modes in contrast to Rayleigh scattering for the smaller particles.

A good microcomposite thermal insulator that minimizes radiative heat transfer should ideally maximize backscattering of the incident thermal radiation to achieve high infrared reflectance, a condition that is characterized by a high *Q*_sca_, and, a low *g* and *Q*_abs_. For a given semiconductor material, these parameters strongly depend on the particle size *d* and the wavelength *λ* of the incident thermal radiation. Thus, we compute the Mie parameters *Q*_sca_, *Q*_abs_ and *g* as a function of particle diameter, from *d* = 0.02 to 3 μm, for wavelengths ranging from *λ* = 0.5 to 10 μm. Furthermore, the spherical microinclusions are assumed to be embedded in an isotropic, non-scattering, non-absorbing and non-magnetic host medium of refractive index *n*_m_ = 1.5. The maxima and minima for *Q*_sca_ and *g* are listed in Tables 1 and 2, respectively, along with their corresponding wavelengths and particle sizes. Table 1 also shows the characteristic bandgap wavelengths *λ*_bg_ for the different semiconductor materials used as microinclusions. Figure 2a–d further shows ${Q}_{{\rm{sca}}}^{{\rm{\max }}}$ and *g*_min_ as a function of the microinclusion size *d* and wavelength *λ*.

**Table 1:** Values for the characteristic bandgap wavelengths *λ*_bg_ (indicated by vertical green arrow-marks in figures), maxima in scattering efficiency ${Q}_{{\rm{sca}}}^{{\rm{\max }}}$ with corresponding wavelengths ${\lambda }_{{Q}_{{\rm{sca}}}^{{\rm{\max }}}}$ and the microcinclusion size ${d}_{{Q}_{{\rm{sca}}}^{{\rm{\max }}}}$ for the different semiconductor materials considered in this study.

Material	*λ*_bg_ ﻿(μm)	${{\boldsymbol{Q}}}_{{\bf{sca}}}^{{\bf{\max }}}$	${{\boldsymbol{d}}}_{{{\boldsymbol{Q}}}_{{\bf{sca}}}^{{\bf{\max }}}}$ (μm)	${{\boldsymbol{\lambda }}}_{{{\boldsymbol{Q}}}_{{\bf{sca}}}^{{\bf{\max }}}}$ ﻿(μm)
InP	0.92	6.3	0.38	0.95
Si	1.11	6.5	0.36	0.97
Ge	1.85	7.5	0.40	1.8
PbS	3.35	7.5	0.74	3.4
InAs	3.44	6.4	1.44	3.8
Te	3.75	10.6	0.68	4.0

**Table 2:** Maxima and minima in the scattering anisotropy *g* for composites with microinclusions of size ${d}_{{g}_{{\rm{\min }}}}$ along with corresponding reflectances ${R}_{{g}_{{\rm{\max }}}}$ and ${R}_{{g}_{{\rm{\min }}}}$ at wavelengths ${\lambda }_{{g}_{{\rm{\max }}}}$ and ${\lambda }_{{g}_{{\rm{\min }}}}$ respectively.

Material	*g*_min_	${{\boldsymbol{d}}}_{{\boldsymbol{g}}{}_{{\bf{\min }}}}$ ﻿(μm)	${{\boldsymbol{\lambda }}}_{{\boldsymbol{g}}{}_{{\bf{\min }}}}$ ﻿(μm)	${{\boldsymbol{R}}}_{{\boldsymbol{g}}{}_{{\bf{\min }}}}$	*g*_max_	${{\boldsymbol{\lambda }}}_{{\boldsymbol{g}}{}_{{\bf{\max }}}}$ ﻿(μm)	${{\boldsymbol{R}}}_{{\boldsymbol{g}}{}_{{\bf{\max }}}}$
InP	−4.94 · 10^−2^	0.60	0.96	0.76	0.547	2.16	0.71
Si	−5.48 · 10^−2^	1.68	2.87	0.63	0.711	1.21	0.49
Ge	−1.19 · 10^−1^	0.64	1.56	0.71	0.524	2.98	0.72
PbS	−1.23 · 10^−1^	1.34	3.26	0.52	0.523	6.30	0.54
InAs	−5.35 · 10^−2^	2.80	4.90	0.50	0.451	6.89	0.57
Te	−2.99 · 10^−1^	1.24	3.92	0.65	0.513	7.85	0.51

**Figure 3:**
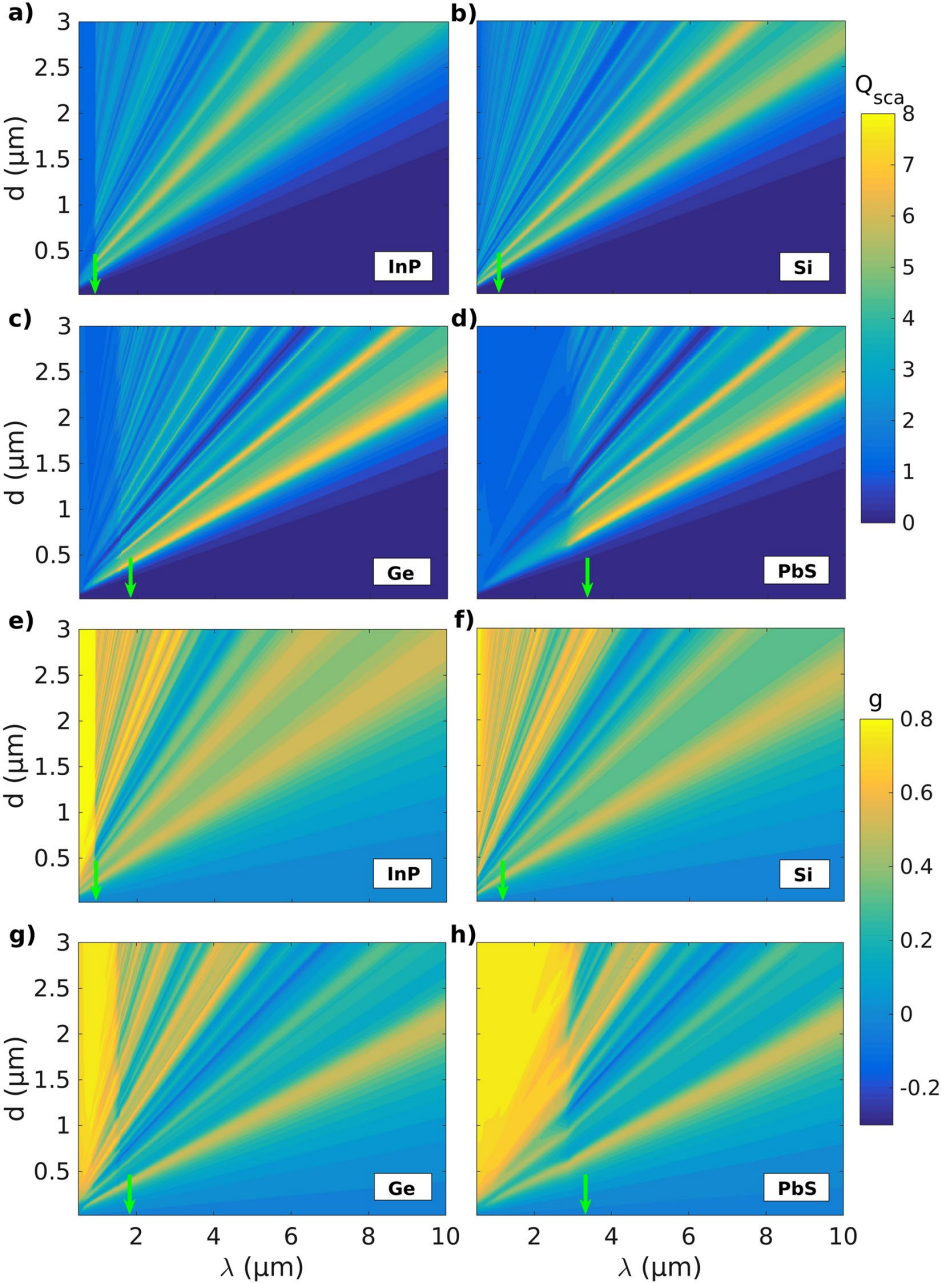
Effect of the change in the wavelength of the incident radiation and the microinclusion diameter on the Mie scattering parameters. (**a**–**d**) Scattering efficiency *Q*_sca_, and, (**e**–**h**) anisotropy factor *g* as a function of the wavelength *λ* of the incident thermal radiation and the diameter *d* of spherical InP, Si, Ge and PbS microinclusions, respectively. The bandgap wavelengths *λ*_bg_ (indicated by vertical green arrow-marks) for the semiconductor materials mark a transition from low to high *Q*_sca_ and strongly forward (+*g*) to mixed scattering regimes for the microinclusions with an increase in *λ*.

Absorption of the incident thermal radiation at wavelengths close to the absorption band-edge (*λ* ≈ *λ*_bg_, Table 1, *λ*_bg_ indicated by vertical green arrow-marks on the x-axis in figures.) gives rise to a significant increase in the number of charge carriers in the conduction (electrons) or the valence band (holes) leading to the excitation of plasmonic resonances in the semiconducting microinclusions. These resonances result in the formation of oscillating multipoles that radiate to generate large values of *Q*_sca_ characterized by broad maxima as shown in Figs 3a–d and SI 4a–b. Figure 3a–d also shows that the maxima in *Q*_sca_ occur when the wavelength of the incident radiation is comparable to the size *d* of the microparticles. For particle sizes *d* ≤ 0.1 μm, *Q*_sca_ remains well below 2.6 for all microinclusion materials and does not attain large values for *λ* ≤ *λ*_bg_ as seen in Fig. 3. This behavior is particularly apparent for composites with PbS (Fig. 3d), InAs and Te (SI Figure 4a–b) microinclusions that have small bandgaps. Figure 2a,c shows that (i) *Q*_sca_ attains a maxima at smaller particle sizes for microinclusions of semiconductors with larger bandgaps or smaller *λ*_bg_ (vertical green arrow-marks), (ii) ${Q}_{{\rm{sca}}}^{{\rm{\max }}}$ is observed to occur at $\lambda \gtrsim {\lambda }_{{\rm{bg}}}$ and, (iii) after the maxima is attained, *Q*_sca_ remains more or less constant with any further increase in particle size. Figure 2b,d further shows that (i) the minima in *g* undergo a sharp switch to negative values upon an increase in particle size beyond a certain limit, (ii) for all materials studied here the negative values for *g*_*min*_ start to occur close to *λ* ≈ *λ*_bg_, and, (iii) the lowest values for *g*_*min*_ are attained immediately after *λ* ≈ *λ*_bg_ with the exception of Si and InAs. In the plots for the forward (*Q*_fs_) and backward (*Q*_bs_) scattering efficiencies corresponding to the particle size ${d}_{{g}_{{\rm{\min }}}}$ it is observed that *Q*_fs_ dominates over *Q*_bs_ (Figs 2e and SI 5). A further deconvolution of the contributions of the different order terms (dipole *n* = 1, quadrupole *n* = 2 and octupole *n* = 3) in Figs 2e and SI 5 indicates that the strongest contributions to *Q*_bs_ arise from the octupole term close to *λ* ≈ *λ*_bg_ followed by the quadrupole and dipole modes at longer wavelengths. Also, particles with sizes comparable to the wavelength of the incident thermal radiation exhibit strong forward scattering (*g* > 0) for *λ* < *λ*_bg_ (Figs 3e–h and SI 4c–d). However, in the limit of Rayleigh scattering the small nanoscale particles exhibit isotropic scattering characterized by *g* values close to zero.

**Figure 4:**
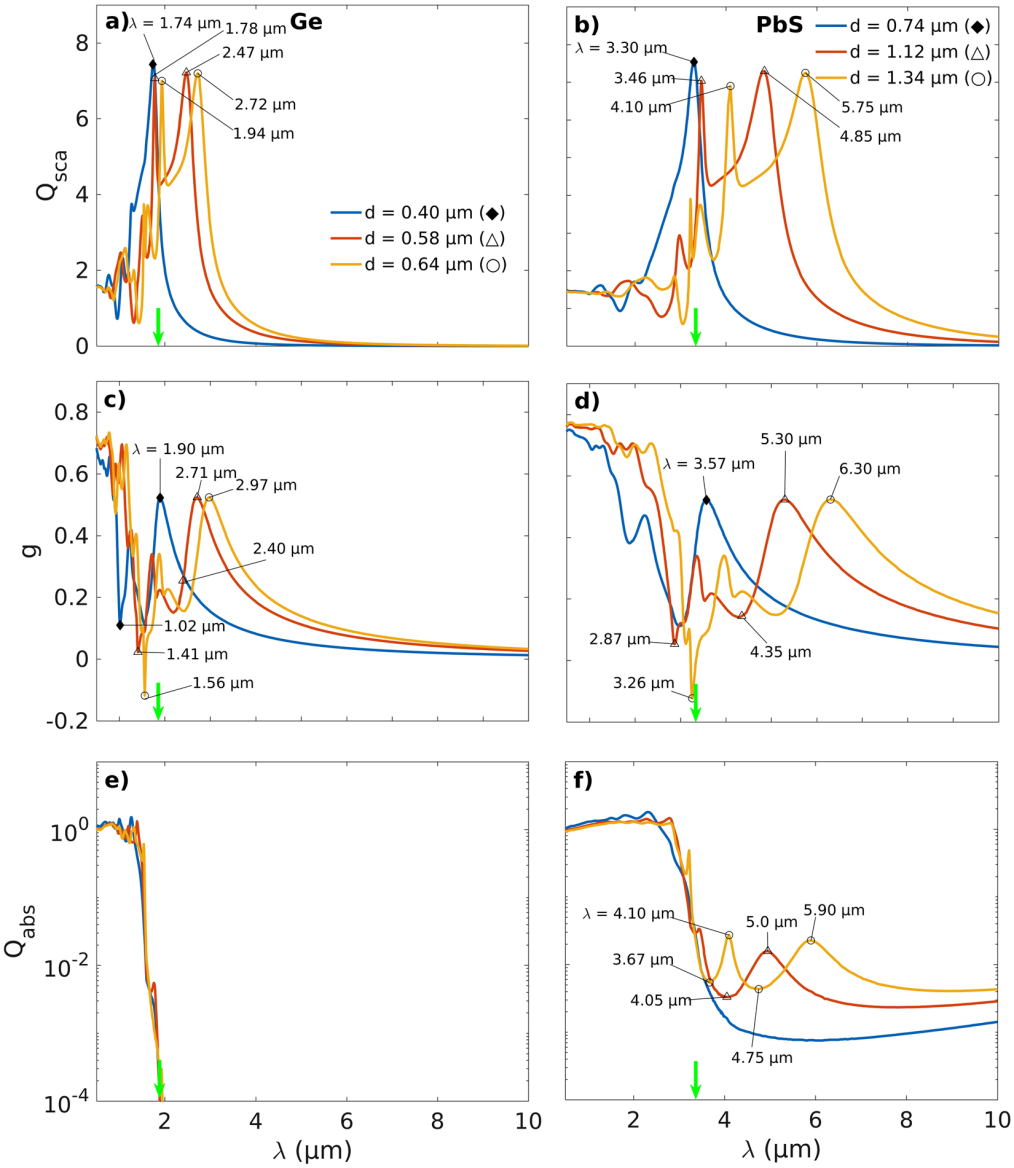
Effect of an increase in the microinclusion size on the Mie parameters. (**a**,**b**) Scattering efficiencies *Q*_sca_, (**c**,**d**) scattering anisotropy *g*, and, (**e**,**f**) absorption efficiencies *Q*_abs_ for various sizes of Ge (left) and PbS (right) microinclusions. The vertical green arrows indicate the bandgap wavelength *λ*_bg_ for the semiconductor materials. A general broadening of the spectral features in *Q*_sca_, *Q*_abs_ and *g* is observed with an increase in the microinclusion size *d*.

Furthermore, it is observed that the local maxima in *Q*_sca_ and plot features in *g* redshift and broaden as the particle size is increased for all semiconducting microinclusion materials considered here (Figs 3, 4a–d, SI 4 and 6). This occurs for increased particle sizes because of a weakening of the restoring force that drives the plasmonic resonances. The restoring force weakens due to an increased distance between the oscillating charges on the opposite sides of a particle leading to a consequent weakening of the interaction between them and hence lower associated energies or a redshift. The effect can be seen more readily when the spectral behavior of *Q*_sca_ and *g* is plotted for Ge and PbS in Fig. 4(a–d) for different particle sizes corresponding to ${Q}_{{\rm{sca}}}^{{\rm{\max }}}$ and *g*_*min*_ shown in Fig. 2 and Tables 1 and 2. For example Fig. 4a shows that the peaks in *Q*_sca_ for Ge at *λ* = 1.78 and 2.47 μm redshift to *λ* = 1.93 and 2.72 μm when the particle size increases from *d* = 0.58 to 0.64 μm (Δ, ○). Similar shifts are observed in *g* for Ge (Fig. 4c), and, *Q*_sca_ and *g* for PbS in Fig. 4b and d respectively.

**Figure 5:**
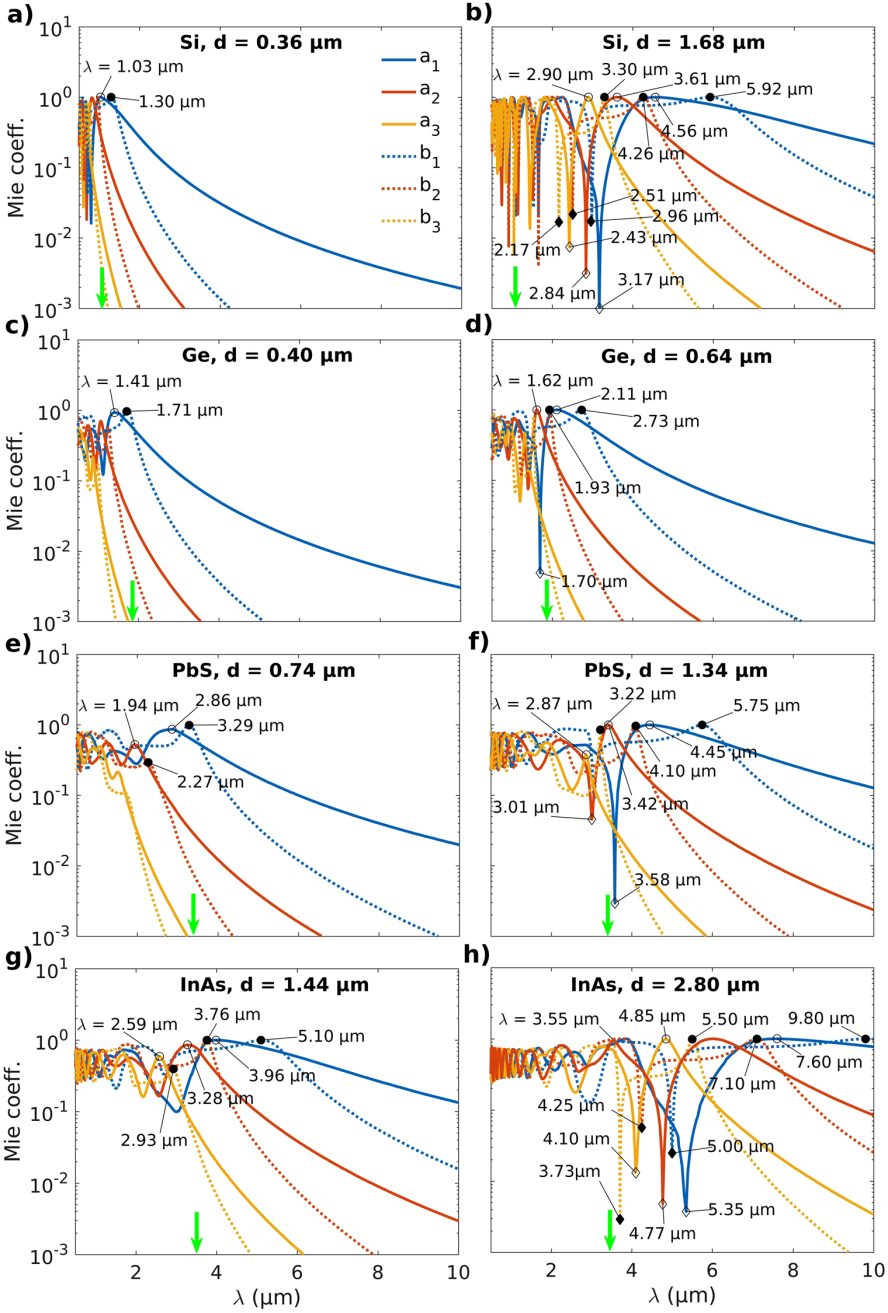
Effect of the semiconductor microinclusion size on the electric and magnetic Mie coefficients. Mie coefficients *a*_*n*_ and *b*_*n*_ as a function of wavelength *λ* for spherical microinclusions of **(a**,**b**) Si, **(c**,**d**) Ge, **(e**,**f**) PbS and **(g**,**h**) InAs for particle diameters *d* corresponding to ${Q}_{{\rm{sca}}}^{{\rm{\max }}}$ and *g*_min_ respectively as shown in Fig. 2. The vertical green arrows indicate the bandgap wavelengths *λ*_bg_. Open and closed symbols denote features in *a*_*n*_ and *b*_*n*_ respectively. An increase in the microinclusion size *d* is accompanied by a strengthening of the magnetic modes *b*_*n*_.

**Figure 6:**
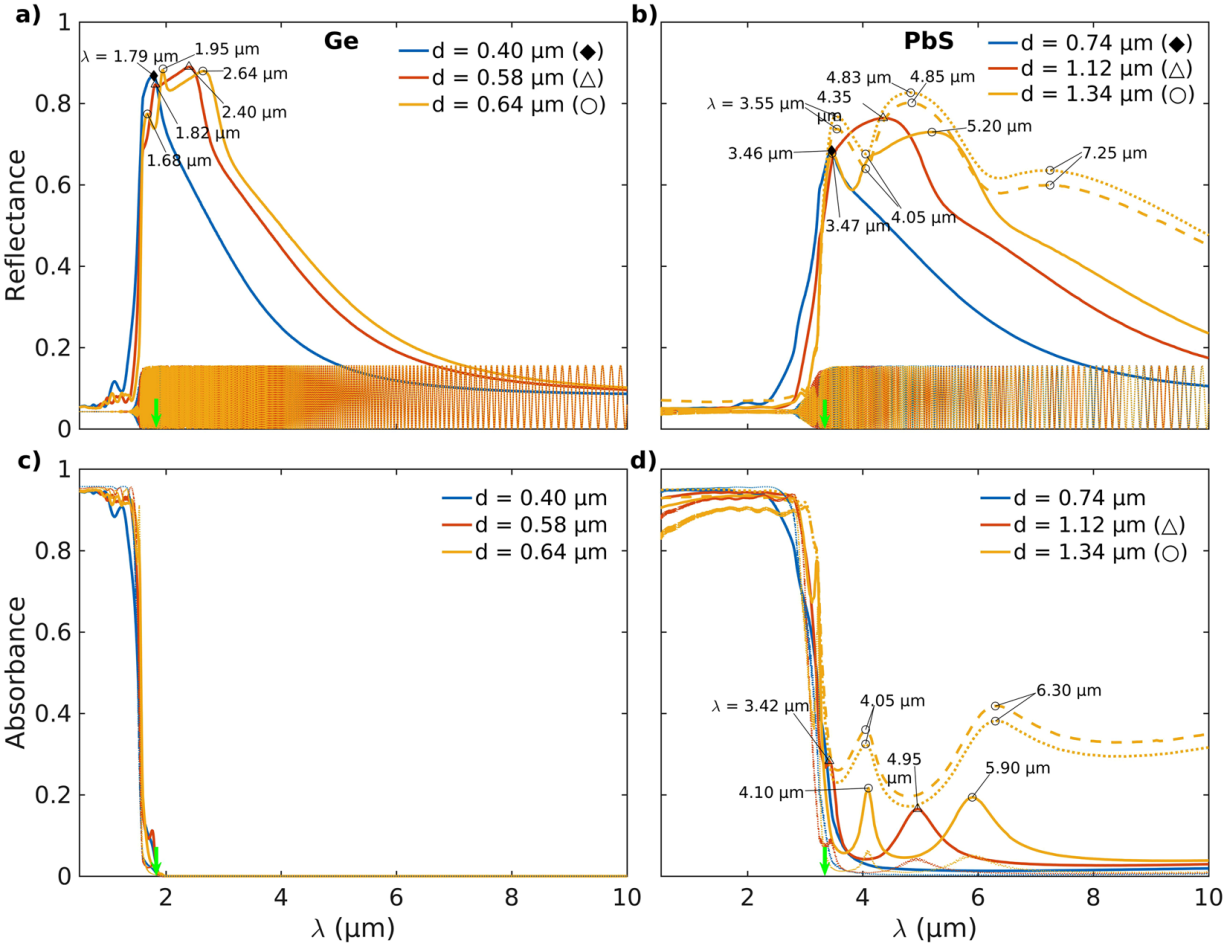
Spectral behavior of insulating composites with low-bandgap semiconductor microinclusions. The spectral reflectance and absorbance of microcomposites with (**a**,**c**) Ge and (**b**,**d**) PbS spherical inclusions of diameter *d* and volume fraction *f* = 0.01, respectively. The solid lines and the thin dotted lines of the same color represent spectral results obtained from the Monte Carlo modeling and Fresnel equations, respectively. In (**b**,**d**), the additional dashed and thick dotted curves in yellow color correspond to results computed using Monte Carlo modeling with a microinclusion volume fraction of *f* = 0.1 and a microcomposite of thickness 2 mm, respectivley. The green arrows indicate the bandgap wavelengths *λ*_bg_. In (**b**,**d**), a broadbanding of the reflectance spectra can be attributed to the plasmonic resonances arising from the collective oscillations of the free charge carriers generated due to weak absorption bands away from the absorption band edge for *λ* > *λ*_bg_.

Figures 5 and SI 7 present the Mie coefficients *a*_*n*_ and *b*_*n*_ for the particles of different semiconductors with sizes *d* corresponding to ${Q}_{{\rm{sca}}}^{{\rm{\max }}}$ and *g*_min_. Compared to the dipole modes, it is observed that the Mie coefficients for the quadrupole and octupole modes decay much faster with increasing wavelength of the incident thermal radiation. As a result, one need only consider the first three modes of the Mie coefficients *a*_*n*_ (○, ◊) and *b*_*n*_ (•, ♦) *i*.*e*. dipole, quadrupole and octupole. Consistent with the features in plots for *Q*_sca_ and *g* (Figs 3, 4, SI 4 and 6), the plasmonic resonances (○, •) are seen to broaden and red-shift with an increase in the semiconductor particle size *d* (Figs 5 and SI 7). Sharp dips (◊, ♦) in the values of the Mie coefficients indicate minima in the extinction efficiency (*Q*_ext_ = *Q*_sca_ + *Q*_abs_) and consequently an increase in transmittance. Results also indicate that the magnetic Mie modes are weaker and decay much faster than the electric modes for all the particle sizes and semiconductor materials considered here (Figs 5 and SI 7). However, consistent with theoretical predictions, a strengthening of the magnetic modes *b*_*n*_ is observed with an increase in the particle size^52^. This strengthening of the magnetic modes is much greater for the Si, PbS, InAs and Te microparticles (Figs 6a,b,e–h and SI 7c–d) compared to that for Ge or InP inclusions (Figs 5c,d and SI 7a–b). Also, the sharp quadrupole and octupole resonances occurring against a background of broad dipole modes for the larger particles give rise to Fano resonances as evidenced by an abrupt switch in the scattering anisotropy *g* from forward (*g* > 0) to backward scattering (*g* < 0) with an increase in particle size (Fig. 2b)^50, 51^.

Further, sharp resonances in *Q*_sca_ for the semiconducting microinclusions can largely be attributed to the points in the spectra where the Mie coefficients *a*_*n*_ (○) and *b*_*n*_ (•) for the electric and magnetic fields, respectively, tend to unity (or maxima), a condition required for the occurrence of scattering resonances^50^. Again, considering Ge and PbS as illustrative examples, it can be seen that there occur Fano resonances in *Q*_sca_ at *λ* = 1.93 and 2.72 μm for Ge particles of size *d* = 0.64 μm (○) (Fig. 4a), and, at *λ* = 4.10 and 5.75 μm for PbS particles of size *d* = 1.34 μm (○) (Fig. 4b). These strong resonances in *Q*_sca_ (Fig. 4a,b) can be attributed to the sharp maxima occuring at the same or close wavelengths in the Mie coefficients *b*_1_ and *b*_2_ corresponding to the magnetic field against a background of the broad contribution to scattering from the electric dipole mode *a*_1_ (Fig. 5c–f). For Si microinclusions of size *d* = 1.68 μm multiple sharp maxima are seen for the dipole, quadrupole and octupole modes for both electric and magnetic Mie coefficients resulting in a large number of Fano resonances in *Q*_sca_ (Figs 5a,b and SI 6b respectively). Similar correspondence between the maxima in *a*_*n*_, *b*_*n*_ and the peaks in *Q*_sca_ occurs for InP (SI Figures 6a and 7a–b), InAs (Figs 5g,h and SI 6c) and Te (SI Figures 6d and 7c–d) microinclusions as well. However, more generally, specific features in *Q*_sca_ and *g* arise from interference effects among the Mie coefficients of different orders.

At the absorption band edge marked by *λ*_bg_ (Table 1), a steep increase in *Q*_abs_ is observed with decreasing *λ* for particles of all materials (Figs 4e–f, SI 8 and 9). The resonances in Mie coefficients *a*_*n*_ and *b*_*n*_ extend beyond *λ*_bg_ for all materials but *Q*_abs_ essentially goes to zero outside the main absorption band, as is to be expected, only for the Ge (Figs 4e and SI 8c), InP and Si microinclusions (SI Figures 8a–b and 9a–b). However, broad peaks in *Q*_abs_ that exist far away from the main absorption band at longer wavelengths and are about 10–20 times weaker are seen for PbS (Fig. 4f), InAs and Te microinclusions (SI Figure 9c–d). These distinctive long-wavelength absorption bands broaden and move farther away from the main absorption band with an increase in the microinclusion size *d*. This is seen in *Q*_abs_ for PbS particles presented in Fig. 4f where these bands with peaks at *λ* = 4.10 and 5.90 μm become distinctive for particles of diameter *d* = 1.34 μm (○). Correspondingly, peaks are also observed in *Q*_sca_ along with associated features in *g* and the Mie coefficients *a*_*n*_ and *b*_*n*_ at close wavelengths, as described earlier (Figs 4b,d and 5e,f, respectively). This, therefore, points to the generation of a sufficiently large number of free charge carriers at *λ* > *λ*_bg_ to enable the generation of plasmonic resonances. Also, it appears that the origin of the weak absorption peaks in *Q*_abs_ for PbS (Fig. 4f), InAs (SI Figure 9c) and Te (SI Figure 9d) microcomposites is likely due to a cluster of defect states within the bandgap with intermediate energies corresponding to the incident thermal radiation. These weak absorption bands at longer wavelengths (*λ* > *λ*_bg_) serve to extend maxima in *Q*_sca_ much beyond the absorption band-edge (Figs 4b and SI 6c,d). However, in the absence of any significant absorption away from the main absorption band (SI Figure 9b), the origin of the several peaks observed in the spectra of *Q*_sca_ for Si microinclusions of size *d* = 1.68 μm (○) is an exception (SI Figure 6b). This may, however, be a result of the complex nature of the band-structure for Si and its indirect bandgap, a discussion of which is beyond the scope of the current article.

### Detailed analysis of Mie scattering in the limit of large and small inclusions

The complicated nature of the formulae for the electric (*a*_*n*_) and magnetic (*b*_*n*_) Mie coefficients (see Methods, Equations 12 and 13) and their complex dependence on the relative refractive index *n*_r_(*λ*), unique to each material, present a challenge to understanding the results for Mie scattering presented in Figs 2, 3, 4, 5 and SI 4–7. However, a great deal of insight into the qualitative behavior for scattering from both large and small particles can be obtained by considering the limiting case of scattering from a small particle. A power series expansion of the spherical Bessel functions in Equations 12 and 13 with respect to the particle size parameter *x* (=*πdn*_m_/*λ*) for the dipolar Mie modes gives^53^

$$\begin{array}{c}{a}_{1}=-i\frac{2{x}^{3}}{3}\frac{{n}_{{\rm{r}}}^{2}-1}{{n}_{{\rm{r}}}^{2}+2}+O({x}^{5}),\\ {b}_{1}=-i\frac{{x}^{5}}{45}({n}_{{\rm{r}}}^{2}-\mathrm{1)}+O({x}^{7})\end{array}$$

with the quadrupole modes given by *a*_2_ ∝ *x* ^5^ + *O*(*x* ^7^) and *b*_2_ = *O*(*x* ^7^). Thus, it can be clearly seen that for $|{n}_{{\rm{r}}}x|\ll 1$ the magnetic dipole mode ${b}_{1}\ll {a}_{1}$, and, it can thus be neglected along with the higher order magnetic and electric modes. Conversely, for |*n*_r_ *x*| ≥ 1 the magnetic modes start to become stronger. This becomes apparent in Figs 5 and SI 7 where a strengthening of the magnetic Mie modes is observed with an increase in the microinclusion size *d*.

For a vanishingly small nonmagnetic particle (*x* → 0) with a finite relative refractive index *n*_r_, the resonance condition in Equation 16 for the electric Mie modes is satisfied when^53^

$${n}_{{\rm{r}}}^{2}({\lambda }_{n})=-\frac{n+1}{n}\mathrm{.}$$

Here, *λ*_*n*_ represents the resonant wavelength for a mode of order *n*. Also, there exists no solution to the corresponding condition (Equation 17) for the magnetic Mie modes. However, since *b*_*n*_ → 0 for vanishingly small particles as shown above one need only consider the resonance condition for the dipole mode *a*_1_ *i*.*e*. *n* = 1. In terms of the dielectric permittivities of the semiconductor particle (*ε*_s_) and the non-absorbing host medium (*ε*_h_) the resonance condition is obtained as

$${\varepsilon }_{{\rm{s}}}^{{\prime} }=-2{\varepsilon }_{{\rm{h}}},\quad {\varepsilon }_{{\rm{s}}}^{{\prime} {\prime} }=0.$$

It is important to note here that the resonance frequencies obtained from the above condition are complex and hence virtual. The real frequency of the incident electromagnetic wave close to where this condition is satisfied for a given material is referred to as the Frölich frequency and the corresponding dipole mode as the Frölich mode. Although the above condition is strictly valid only for a vanishingly small particle, the frequency shift for the Frölich mode as a function of an increasing size parameter *x* can be understood through an expansion of the spherical Bessel and Hankel functions in Equation 16 into a power series in *x*. This gives^53^

$${\varepsilon }_{{\rm{s}}}=-\mathrm{(2}+12{x}^{2}\mathrm{/5)}{\varepsilon }_{{\rm{h}}}$$

Now, for all semiconductor materials considered here, largely an increase in the real part of the dielectric permittivity ${\varepsilon }_{{\rm{s}}}^{{\prime} }$ is observed with a decrease in the wavelength *λ* except for the very short wavelengths deep into the absorption band (SI Figure 3). Thus, an increase in the size parameter *x* or the particle size shifts the Frölich frequency to lower values or longer wavelengths. This redshift of the scattering resonance peaks with an increase in the particle size is clearly seen in Figs 3, 4, 5 and SI 5–7 for all low bandgap semiconductor materials used here in our study.

The deconvolution of the back scattering efficiency (*Q*_*bs*_) into the contributions from the octupole, quadrupole and the dipole modes in Figs 2e and SI 5 shows anomalous scattering with an inverse hierarchy of scattering resonances in contrast to Rayleigh scattering wherein the dipole mode is the strongest. This is a direct consequence of the (2*n* + 1) factor that occurs in the equations for the forward and back scattering efficiencies (Equations 19 and 20) when the size parameter $x({\lambda }_{n})\gtrsim 1$. Here, the size parameter for the resonant modes varies between *x*_*n*_ ∈ [0.83, 2.55] wherein the lower limit corresponds to the dipole mode (*λ*_*n*_ = 7.05 μm) in Te microinclusions while the upper limit corresponds to the octupole mode (*λ*_*n*_ = 1.11 μm) in InP microinclusions. Further, this anomalous scattering is known to occur when the radiative damping dominates over the dissipative losses in a material *i*.*e*.^54^

$${\varepsilon }_{s}^{{\prime\prime} }({\lambda }_{n})\ll \frac{{x}_{n}^{2n+1}}{n{\mathrm{[(2}n-\mathrm{1)!!]}}^{2}}$$

SI Table 1 presents a comparison of the results for the radiative damping term on the right for the strongest of the dipole, quadrupole and the octupole modes (Figs 2e and SI 5) with the dissipative term on the left ${\varepsilon }_{s}^{{\prime\prime} }$ (=2*η*_*s*_ *κ*_*s*_, SI Figure 3b) in the above inequality. It is seen that for the InP and Si microinclusions this condition is readily satisfied for all the three modes while for Ge microinclusions only dipole and quadrupole modes satisfy this condition strongly. However, the microinclusions of PbS, InAs and Te satisfy these conditions only weakly for the various modes. In all these materials, the radiative damping associated with the dipole modes is observed to be the strongest followed by the quadrupole and the octupole modes.

### Spectral reflectance of microcomposites

This section presents results on the spectral characteristics of composites with low-bandgap semiconductor microinclusions computed using Monte Carlo modeling and Fresnel equations. For Monte Carlo modeling, we employ the spherical microinclusions of optimal size *d* determined using Mie theory for obtaining maximum *Q*_sca_ and minimum *g* for the various semiconductor materials (Table 1). Furthermore, for all our computations here, we consider a microcomposite with a thickness *t* = 200 μm and a semiconductor microinclusion volume fraction of *f* = 0.01 unless specified otherwise. Considering a cylindrical symmetry for the propagation of the infinitesimally thin beam of incident thermal radiation in the Monte Carlo model, a grid resolution of *dz* = 2 μm and *dr* = 1 μm is used for the radial $\hat{r}$ and axial $\hat{z}$ directions respectively (see Fig. 1). The total number of grid elements in the $\hat{r}$-direction is set to *N*_*r*_ = 100 while the number of grid elements *N*_*z*_ in the $\hat{z}$-direction is determined by the thickness of the microcomposite layer. Adequate care is also taken to ensure that the diffuse reflectance and transmittance go to zero as a function of the radius *r* while their angular dependence on the photon-exiting direction $\hat{\alpha }$ is ignored. To compute the infrared spectra for the incident thermal radiation, 10^7^ photons are launched for each wavelength *λ* considered.

**Figure 7:**
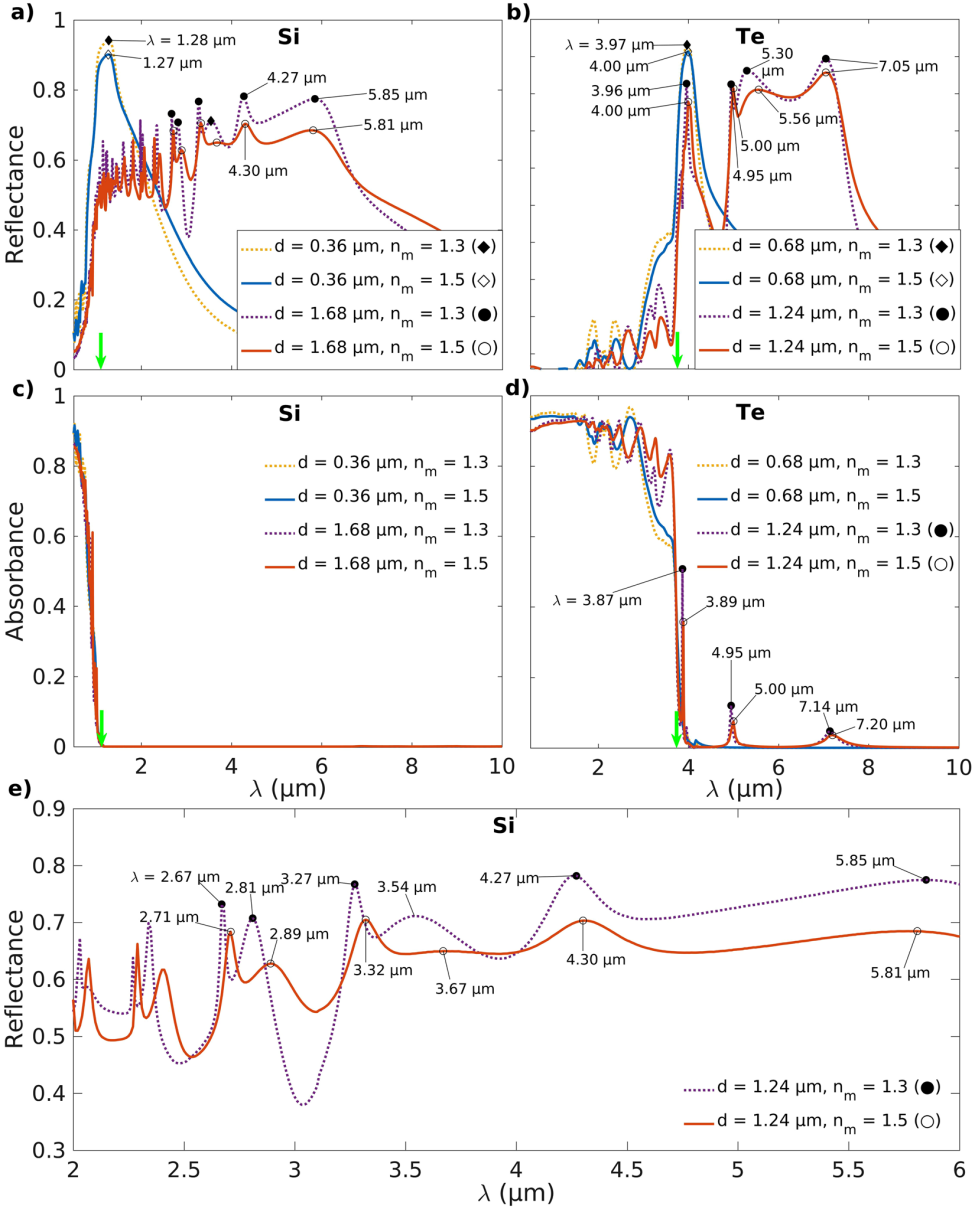
Effect of an increase in the refractive index of the host medium on the optical spectra of the microcomposites due to plasmonic resonances. (**a**,**b**) Spectral reflectance and (**c**,**d**) absorbance for microcomposites with a volume fraction *f* = 0.01 of (**a**,**c**) Si and (**b**,**d**) Te particles of different sizes *d* embedded in a dielectric medium of refractive index *n*_m_ = 1.5 and 1.3. (**e**) An expanded view of the reflectance peaks for composites with Si microinclusions shown in (**b**). A clear redshift in the reflectance peaks is observed with an increase in the refractive index of the host medium pointing to the generation of LSPRs in the larger semiconductor microinclusions.

Figures 6 and 7 show the reflection and the absorption spectra for infrared radiation ranging from *λ* = 0.5 to 10 μm for composite layers with Ge and PbS, and, Si and Te microinclusions, respectively. A comparison of the results from Monte Carlo modeling and Fresnel equations for radiation transport clearly shows that the presence of the low-bandgap semiconducting microinclusions significantly increases both the reflectance and the absorbance of the microcomposite layers (Figs 6 and SI 10). This is because, unlike Fresnel equations, the Monte Carlo model takes into account the plasmonic resonance induced enhanced scattering from the microparticles. This results in a decreased mean free path (∝[*μ*_abs_ + *μ*_sca_]^−1^) and diffusive transport of the incident radiation in the microcomposite layer thereby giving rise to greater absorbance and reflectance. For a host medium refractive index of *n*_m_ = 1.5, among the semiconductor materials considered, the highest reflectance *R* = 0.91 is obtained for Te microcomposites at *λ* = 4.0 μm for microinclusions of size *d* = 0.68 μm (♦) (Fig. 7b). A similar value of *R* = 0.90 is also obtained for the Si microcomposites at *λ* = 1.27 μm for inclusions of diameter *d* = 0.36 μm (♦) (Fig. 7a). Furthermore, for microcomposites with Ge inclusions of diameter *d* = 0.64 μm (○) (Fig. 6a), two high peaks (*R* ≈ 0.88) in the reflectance ocurring at *λ* = 1.95 and 2.64 μm can be directly attributed to the peaks in *Q*_sca_ at *λ* = 1.94 and 2.72 μm (Fig. 4a). On the other hand, the reflectance calculated using Fresnel equations for all microcomposites remains well below *R* = 0.2 (Figs 6a–b and SI 10a–b). This difference between the results from Monte Carlo modeling and Fresnel equations emphasizes the hugely disproportionate impact a small volume fraction of microparticle inclusions makes on the infrared spectra of the micromposite layer. Additionally, they also underline the importance of considering scattering from particles that are comparable in size to the wavelength *λ* of the incident radiation.

The peaks in reflectance are seen to redshift and broaden by various amounts for the different microcomposites with an increase in the size *d* of the particle inclusions (see Figs 6a,b, 7a,b and SI 10a–b). The effect is observed to be especially pronounced for composites with PbS, Si, Te and InAs microinclusions (Figs 6b, 7a,b, SI 10b, respectively). This broadbanding of the reflectance spectra is a direct consequence of the red-shifting and broadening of the sharp Fano resonances for larger microinclusions in the spectra for *Q*_sca_ (Figs 4b and SI 6b–d). In PbS, Te and InAs microcomposites (Figs 6b, 7b and SI 10b, respectively), the broadbanding of the reflectance for the larger microinclusions appears to be driven, in part, by the enhanced scattering from plasmonic resonances generated due to the presence of weak absorption peaks far outside the main absorption band (Figs 6d, 7d and SI 10d, respectively). It is notable in this regard that Felts *et al*.^55^ have experimentally observed LSPRs in silicon-doped InAs microparticles of size 1.0 μm with characteristic absorbance at wavelengths *λ* = 5.75 and 7.70 μm. The wavelengths at which these LSPR-associated absorbance maxima occur are similar to the wavelengths we observe for the absorbance maxima in composites with InAs microinclusions at *λ* = 5.55 and 7.15 μm (○), and, *λ* = 5.35 μm (◊) for particles of size *d* = 2.80 and 1.44 μm, respectively (*n*_m_ = 1.5, SI Figure 10d). Additionally, it is also observed that microcomposites with larger inclusions exhibit lower maxima in reflectance (Figs 6a,b, 7a,b and SI 10a–b), although the maxima in *Q*_sca_(*λ*, *d*) remain approximately constant with any further increase in *d* after they reach a peak value (Fig. 2a). This happens because, for a given volume fraction *f*, the scattering coefficient *μ*_sca_ in Equation (3) is directly proportional to *Q*_sca_ but scales inversely with *d*.

In all the microcomposites studied here, plasmonic resonance driven peaks in reflectance spectra (Figs 6a,b, 7a,b, SI 10a–b) appear right before the absorption band edge due to low characteristic values of *Q*_abs_ for wavelengths $\lambda \gtrsim {\lambda }_{{\rm{bg}}}$ (Figs 4e,f, SI 8 and 9). This is regardless of whether there exists a maxima in *Q*_sca_(*λ*, *d*) or not in that wavelength range for a given microinclusion size. This is illustrated by microcomposites with PbS particles of diameter *d* = 1.34 μm (○) that present a peak in reflectance with *R* = 0.68 at *λ* = 3.47 μm in Fig. 6b despite the moderate *Q*_sca_ = 3.63 and a value of *g* = 7.63 · 10^−2^ pointing to isotropic scattering (Fig. 4b,d). On the other hand, comparable scattering parameters *Q*_sca_ = 3.90 and *g* = 3.60 · 10^−2^ at *λ* = 3.22 μm (Fig. 4b,d) suggest higher reflectance although the actual observed reflectance *R* = 0.24 is quite low compared to *R* = 0.68 (Fig. 6b). Still, a significant change in reflectance occurs due to the absorption efficiency decreasing from *Q*_abs_ = 0.48 at *λ* = 3.22 μm to a low value of *Q*_abs_ = 8.70 · 10^−2^ at *λ* = 3.47 μm (Fig. 4f).

Figure 6a,b shows that the reflectance values *R* = 0.71, 0.52 associated with Ge and PbS microinclusions of size *d* = 0.64, 1.34 μm and corresponding to the minima in scattering anisotropy *g*_min_ = (−1.19, 1.23) · 10^−1^ at *λ* = 1.56, 3.26 μm, respectively (Fig. 2b, Table 2), are not the highest values of reflectance obtained for both Ge and PbS. In the case of Ge and PbS microinclusions, this is in part explained by the fact that the wavelengths ${\lambda }_{{g}_{{\rm{\min }}}}$ (Table 2) corresponding to *g*_min_ (Fig. 4c,d) are located within the main absorption band (Table 1) wherein *Q*_sca_ is low (Fig. 4a,b) and *Q*_abs_ is high (Fig. 4e,f). Furthermore, both Ge and PbS microinclusions of sizes *d* = 0.58, 1.12 μm (Δ) are found to be forward-scattering for the reflectance maxima at *λ* = 2.40, 4.35 μm (Fig. 6a,b) with scattering anisotropy *g* = 0.25, 0.14 (Fig. 4c,d), respectively, thereby implying that a low value of the scattering anisotropy *g* is not essential to obtain high reflectance. Composites with InAs microinclusions of size ${d}_{{g}_{{\rm{\min }}}}=2.80$ μm show a reflectance ${R}_{{g}_{{\rm{\max }}}}=0.57$ that is higher than the reflectance ${R}_{{g}_{{\rm{\min }}}}=0.50$ (Table 2). On the other hand, as per expectations, composites with InP, Si and Te microinclusions of size ${d}_{{g}_{{\rm{\min }}}}$ exhibit a higher reflectance ${R}_{{g}_{{\rm{\min }}}}$ than ${R}_{{g}_{{\rm{\max }}}}$ (Table 2). Thus, there appears to be scant correlation between a low negative value for the scattering anisotropy *g* and a high value of reflectance *R* due to the conflicting evidence presented by the results for the microinclusion materials considered here. This is likely because once a photon is launched into a highly scattering microcomposite layer, early on during its motion, the direction of propagation of the photon gets quickly randomized. As a consequence, a low negative value of the scattering anisotropy *g* is rendered rather ineffective compared to the stronger influence of the scattering (*Q*_sca_) and absorption (*Q*_abs_) efficiencies.

Figure 6b,d shows the reflectance and absorbance spectra for the microcomposites with PbS microinclusions of diameter *d* = 1.34 μm (○) for two different volume fractions *f* = 0.01 (*t* = 200 μm and 2 mm) and 0.1. It is observed that the increase in volume fraction from *f* = 0.01 to 0.1 shifts the peak in reflectance at *λ* = 5.20 μm to *λ* = 4.85 μm and results in a new reflectance peak at *λ* = 7.25 μm. The peak at *λ* = 7.25 μm also appears in the reflectance for the microcomposite with a PbS particle volume fraction *f* = 0.01 and thickness *t* = 2 mm. More generally, this implies that a larger number of particles is required to produce enough scattering to reflect the longer wavelength infrared radiation because a microcomposite of thickness *t* = 200 μm and volume fraction *f* = 0.01 has only 1/10^*th*^ the number of particles compared to the other two microcomposites with increased thickness (*t* = 2 mm) and volume fraction (*f* = 0.1), respectively.

An increase in the volume fraction *f* of the low-bandgap semiconducting microinclusions increases scattering and hence has the general effect of increasing the reflectance *R* of the microcomposite. However, beyond a point any further increase in *f* to increase *R* is counteracted by an increase in the absorbance that would be detrimental to the performance of an insulating microcomposite. This is evident from Fig. 6b wherein the reflectance at *λ* = 4.05 μm for a PbS microcomposite decreases from a value of *R* = 0.68 for *f* = 0.01 (*t* = 200 μm) to *R* = 0.64 for *f* = 0.1 (*t* = 200 μm).

### Nature of plasmonic resonances

Plasmonic resonances observed in the semiconductor microinclusions can have both surface and volume modes with contributions from the magnetic or electric Mie coefficients (*a*_*n*_ or *b*_*n*_) or both. A key feature of the surface modes or LSPRs is the broadening and red-shifting of the scattering resonances with an increase in the particle size *d* ^56^. This is seen clearly manifested to varying degrees in the Mie scattering efficiencies *Q*_sca_ for the various semiconductor microinclusion materials considered here (Figs 3a–d, 4a,b, SI 4a-b and 6). Additionally, LSPRs are also known to exhibit a red-shift with an increase in the refractive index of the host medium^56–58^. Thus, to ascertain further the nature of the plasmonic resonances observed in the spectra for the different microcomposites, we compare and contrast the optical spectra obtained using host refractive index *n*_m_ = 1.5 (○, ◊) with the results from *n*_m_ = 1.3 (•, ♦). Figure 7a,b shows that for composites with the larger Si and Te microinclusions there occurs a red-shift in the reflectance peaks with an increase in the refractive index of the host medium while for the smaller particles such a change is not clearly discernible. Reflectance peaks at *λ* = 4.27, 3.54, 3.27, 2.81 and 2.67 μm in the spectra for composites with Si microinclusions of size *d* = 1.68 μm red-shift to *λ* = 4.30, 3.67, 3.32, 2.89 and 2.71 μm respectively with a change in the host medium refractive index from *n*_m_ = 1.3 (•) to 1.5 (○) (Fig. 7e). For composites with Te microinclusions of size *d* = 1.24 μm reflectance peaks shift from *λ* = 5.30, 4.95 and 3.96 (•) to *λ* = 5.56, 5.00 and 4.00 μm (○) respectively for this change in the refractive index of the host medium. The notable exceptions to this red-shift occur for the broad peaks at longer wavelengths *λ* ≈ 5.8 and 7.0 μm for composites with Si (*d* = 1.68 μm) and Te (*d* = 1.24 μm) microparticles, respectively. The likely cause for this could either be that these peaks are associated with plasmonic resonances that are volume modes or the red-shift is masked due to the broadness of the peaks. A similar trend in the red-shifting of the peaks in the reflectance spectra associated with larger microinclusion size and a change in the refractive index of the host medium is generally observed in composites with InP, InAs, Ge, and PbS microinclusions as well (SI Figures 10a–b and 11a–b, respectively). In the case of microcomposites with PbS, InAs and Te inclusions, the weak plasmonic absorption peaks that are associated with reflectance maxima outside the main absorption band exhibit similar redshift with an increase in the host refractive index (SI Figures 10d and 11d, and, Fig. 7d respectively). Thus, there appears to be a transformation in the nature of the plasmonic resonances from volume modes for the smaller microinclusions to LSPRs for composites with the larger semiconductor microinclusions. Also, it is apparent from the results presented earlier for Mie scattering that this shift is associated with and driven by a strengthening of the magnetic modes *b*_*n*_ characteristic of the larger particles (Fig. 5). For the large spherical microinclusions considered here, these resonances can thus be connected to oscillatory eddy currents generated by electromagnetic waves traveling large distances along the surface of the particles^59, 60^.

### Reflectance efficiency of the microcomposites

**Figure 8:**
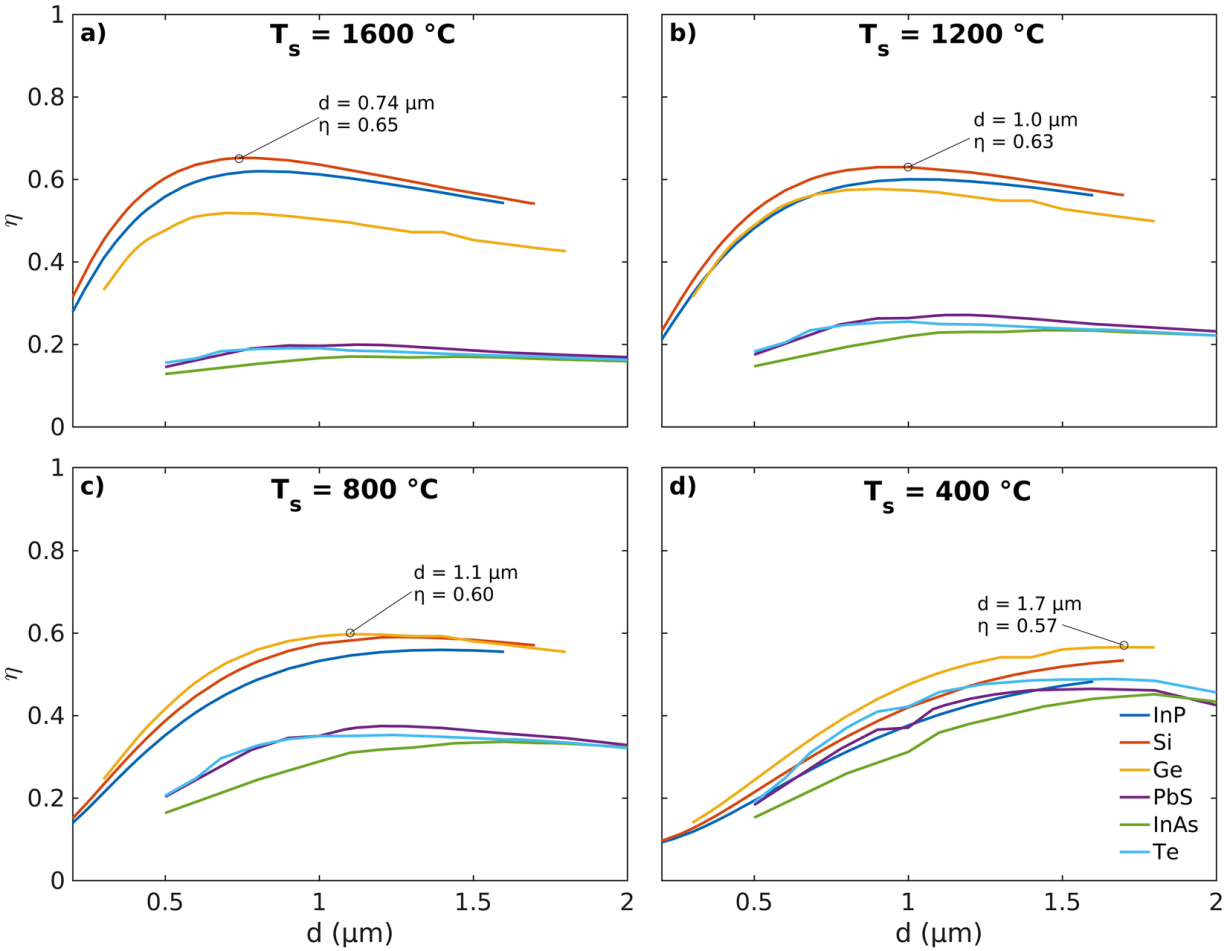
Effect of microinclusion size and low-bandgap semiconductor material on the reflectance efficiency of insulating microcomposites. Reflectance efficiencies *η* of microcomposites with InP, Si, Ge, PbS, InAs and Te microinclusions for incident blackbody radiation from sources at temperatures in the range 400 ≤ *T*_s_ ≤ 1600 °C. The semiconductor microinclusions that have their bandgap wavelengths *λ*_bg_ close to or slightly greater than the wavelength *λ*_max_ of the peak spectral radiance from a blackbody source are the most effective in maximizing reflectance of the incident heat radiation.

To assess the effectiveness of the different microcomposite materials in preventing thermal losses through radiative transfer, the reflectance efficiency *η*(*λ*, *d*), defined in equation (22) (see Theory and Methods), is computed as a function of the size *d* of the semiconducting microinclusions. The calculations for *η* cover the entire wavelength range of interest (*λ* = 0.5 to 10 μm) for the incident radiation from blackbody sources at temperatures *T*_s_ = 1600, 1200, 800 and 400 °C. Here, we note that the peak spectral radiance for a blackbody at temperatures *T*_s_ = 1600, 1200, 800 and 400 °C is obtained at *λ*_max_ = 1.55, 1.97, 2.70 and 4.31 μm respectively. Figure 8 shows high values of (0.65 > *η* > 0.55) implying reflectances of over 60% obtained from microcomposites with an optimal size *d* of the semiconducting microinclusions. For the blackbody radiation from sources at temperatures *T*_s_ = 1600 and 1200 °C, the highest values of efficiency *η* = 0.65 and 0.63 are obtained for Si microcomposites with optimal microinclusion diameters *d* = 0.74 and 1.0 μm respectively (Fig. 8a,b). On the other hand, microcomposites with Ge inclusions of optimal diameters *d* = 1.10 and 1.70 μm attain the highest efficiency values of *η* = 0.60 and 0.57 for radiation sources characterized by temperatures *T*_s_ = 800 and 400 °C respectively (Fig. 8c,d). These results thus show that as the wavelength *λ*_max_ for the peak spectral radiance increases with decreasing source temperatures, the size of the microinclusions required for obtaining peak reflectance efficiency also increases. This shift in the optimal particle diameters *d* for obtaining maximal reflectance efficiency *η* is consistent with the broadening and shifting of the peaks for *Q*_sca_ (Figs 3a–d, 4a–b, SI 4a–b and 6) and reflectance *R* (Figs 6a,b, 7a,b and SI 10a–b) towards longer wavelengths with increasing microinclusion size *d*.

Among all the semiconducting materials considered here, it is also observed that Si, Ge and InP microinclusions with larger bandgaps are the only effective inclusion materials for incident blackbody radiation from sources at temperatures in the range 400 ≤ *T*_s_ ≤ 1600 °C (Fig. 8). It is observed that these materials are characterized by a strong radiative damping for all three Mie modes (*n* = 1, 2 and 3) and low dissipation (${\varepsilon }_{{\rm{s}}}^{{\prime\prime} }$(*λ*_*n*_)) in contrast to PbS, InAs and Te microinclusions (SI Table 1 and SI Figure 3b). Furthermore, Fig. 8 shows that the three semiconductors (PbS, InAs and Te) with the smaller bandgaps begin to significantly contribute to the reflectance efficiency *η* only when their corresponding bandgap wavelength *λ*_bg_ becomes smaller than the wavelength (*λ*_max_ = 4.31 μm) for the peak spectral radiance corresponding to the lowest source temperature *T*_s_ = 400 °C (Fig. 8d). Composites with Te microinclusions exhibit the most promising set of Mie parameters, a high *Q*_sca_ and the most negative *g*_min_ (Tables 1 and 2), and the highest peak in reflectance *R* = 0.91 observed amongst all the semiconducting microinclusions (Fig. 7b). However, despite this, a high reflectance efficiency *η* is not observed in Te microcomposites for any of the blackbody source temperatures *T*_s_ considered here because of the large bandgap wavelength *λ*_bg_ = 3.75 μm for Te (Table 1). In contrast, InP, Si and Ge have bandgaps occurring at wavelengths *λ*_bg_ = 0.92, 1.11 and 1.85 μm (see Table 1) that are all smaller than the wavelengths *λ*_max_ for the peak spectral radiance of the blackbody source temperatures considered here. Thus, it can be inferred that semiconducting microinclusions with their bandgap wavelengths *λ*_bg_ close to or slightly greater than the wavelength *λ*_max_ of the peak spectral radiance from a blackbody source are the most effective in maximizing reflectance. This happens because close to the wavelength *λ*_bg_ there exist enough free charge carriers in the conduction or valence band to allow for the excitation of LSPRs that improve reflectance of the incident thermal radiation through enhanced scattering.

## Conclusions

To summarize, we have investigated the use of plasmonic resonance driven enhanced scattering from low-bandgap semiconductor microinclusions for tailoring the spectral properties of insulating composites to prevent radiative thermal losses in high temperature applications. To simulate radiative transfer in composites with semiconductor microinclusions of different materials, we have employed Monte Carlo modeling in conjunction with Mie theory. We have also compared and contrasted our results from the Monte Carlo modeling with reflectance and absorbance spectra obtained from Fresnel’s equations, based on MG-EMT, that do not account for scattering from the microinclusions. Comparative results show that there is a significant enhancement in reflectance and absorbance of the incident thermal radiation due to a decrease in the average pathlength of the photons in the microcomposite layer from enhanced scattering.

The key focus of our effort in this study has been to understand the role of the size-dependent Mie scattering (*Q*_sca_) and absorption (*Q*_abs_) efficiencies and the scattering anisotropy *g* of microinclusions in maximizing the thermal reflectance efficiency *η*. Our results show that Mie coefficients of order *n* ≤ 3 alone contribute significantly to the Mie parameters for the spherical microinclusions. The Mie coefficients *a*_*n*_ and *b*_*n*_ corresponding to the electric and magnetic fields, respectively, show that the spectral features in *Q*_abs_, *Q*_sca_ and *g* arise from the interference effects among different multipole contributions. The sharp peaks in the higher order magnetic modes for the larger microinclusions against a background of the broad dipole modes give rise to Fano resonances that generate sharp peaks in the scattering efficiency *Q*_sca_. For all semiconducting microinclusions, the first of the plasmonic resonance driven peaks in reflectance appear just outside the absorption band edge for wavelengths $\lambda \gtrsim {\lambda }_{{\rm{bg}}}$. The spectral features in *Q*_sca_ and *Q*_abs_ redshift and broaden with an increase in the size *d* of the semiconducting microinclusions caused by an increase in the strength of the magnetic modes *b*_*n*_. This redshift and broadening of spectral features is also seen in the reflectance and absorbance spectra for the different semiconducting materials used as inclusions in the insulating dielectric. For some semiconductor microinclusions (PbS, Te and InAs) a further broadbanding of the reflectance spectra is observed to be associated with absorbance peaks that are about 10–20 times weaker as compared to the main absorption band. These absorbance peaks likely arise due to defect states within the bandgap that contribute enough charge carriers to the conduction or the valence band for plasmonic resonance driven enhanced scattering resulting in increased reflectance. A redshift in the reflectance peaks for the larger microinclusions with an increase in the refractive index of the host medium points to the transformation in the nature of the plasmonic resonances from volume modes for the smaller particles to LSPRs for the larger microinclusions. A low negative value of the scattering anisotropy *g* lying outside the main absorption band does appear to enhance reflectance as hypothesized, but the resulting effect is not as pronounced as that from changes in *Q*_sca_ and *Q*_abs_. A high value of reflectance *R* ≥ 88% observed in the spectra, for the different semiconducting microinclusions considered here, is in general associated with high scattering and low absorption efficiencies obtained from Mie theory.

An increase in the volume fraction *f* of the microinclusions or an increase in the thickness *t* of the microcomposite lead to broadening of the reflectance at longer wavelengths that is often accompanied by an appearance of additional peaks. Results for the reflectance efficiency *η* show that semiconducting microinclusions (Si, Ge and InP) with their bandgap wavelengths (*λ*_bg_) close to and greater than the wavelength (*λ*_max_) of the peak spectral radiance for incident blackbody radiation from a source at a given temperature *T*_s_ serves to maximize *η*. The highest reflectance efficiencies 0.57 ≤ *η* ≤ 0.65, corresponding to more than 57% back-reflectance, are obtained for Si and Ge microinclusions at really low volume fractions (*f* = 0.01) for incident blackbody radiation from sources at temperatures in the range 400 ≤ *T*_s_ ≤ 1600 °C. The high reflectance efficiency of composites with Si and Ge microinclusions is also seen to be correlated to the strong radiation damping and low dissipation at wavelengths *λ*_*n*_ corresponding to the dipole, quadrupole and for radiation sources characterized octupole resonances in the scattering efficiency *Q*_sca_. It is also observed that with an increase in the wavelength (*λ*_max_) for the peak spectral radiance a commensurate increase in the size of the semiconducting microinclusions is also required for obtaining optimal reflectance efficiency *η*. Thus, to fully maximize reflectance for preventing thermal losses through radiative transfer, polydispersity in the size of the microinclusions is desirable.

In conclusion, we have demonstrated that enhanced scattering due to plasmonic resonances in low-bandgap semiconductor microinclusions at really small volume fractions in an insulating dielectric can be exploited for preventing radiative thermal losses by maximizing reflectance of the incident infrared radiation in high temperature applications. Our results also suggest that the use of semiconductor microinclusions in insulating dielectrics offers a possiblity for the further enhancement and broadbanding of the reflectance spectra through the use of dopants for engineering defect states within the semiconductor bandgap that contribute to LSPRs at thermal infrared wavelengths.

## Theory and Methods

### Monte Carlo Model

For modeling thermal radiative transfer in an insulating dielectric with randomly dispersed low-bandgap semiconducting microparticles we employ a Monte Carlo method primarily developed and designed by Wang *et al*. for modeling radiation transport in turbid media^39^. To isolate the role of plasmonic resonance driven scattering in enhancing diffuse reflectance, we make the simplifying assumption that the semiconductor microparticles are embedded in an isotropic, non-scattering and non-absorbing host material with an effective dielectric constant *ε*_h_ = 2.25. Additionally, the dielectric microcomposite layer is characterized by a thickness *t*, effective refractive index *n*_L_, absorption coefficient *μ*_abs_, scattering coefficient *μ*_sca_ and a scattering anisotropy factor *g*. The composite layer is also assumed to be free-standing in a medium with a dielectric constant of *ε*_0_ = 1.

Briefly, the Monte Carlo method models radiative thermal transport by tracking packets of energy or photons launched perpendicularly into the composite layer (See Fig. 1 for a schematic). Each photon is characterized by a weight factor that is initialized to unity before its launch. Once a photon enters the microcomposite layer, the step size *s* for its propagation is given by

1 $$s=-\frac{\mathrm{ln}(\xi )}{{\mu }_{{\rm{abs}}}+{\mu }_{{\rm{sca}}}}\mathrm{.}$$

Here, *ξ* is a random variable uniformly distributed over the interval (0, 1). If during propagation the photon hits a boundary between two dissimilar media then the probability *R* of it being reflected back is defined to be an average of the reflectances for the two orthogonal polarizations

2 $$R=\frac{1}{2}(\frac{{\sin }^{2}({\varphi }_{0}-{\varphi }_{1})}{{\sin }^{2}({\varphi }_{0}+{\varphi }_{1})}+\frac{{\tan }^{2}({\varphi }_{0}-{\varphi }_{1})}{{\tan }^{2}({\varphi }_{0}+{\varphi }_{1})}),$$

to account for the unpolarized nature of the incident and propagating thermal radiation. Here, *ϕ*_0_ and *ϕ*_1_ are the angles of incidence and transmittance, respectively. If the photon does not hit a boundary, its weight is decremented by the fraction of the energy absorbed in the microcomposite. A new direction is then sampled according to the Henyey-Greenstein function^61^ using the scattering anisotropy *g*. The values for *g* vary between −1 and +1 with the upper and lower limits corresponding to totally asymmetric backward and forward scattering, respectively. The photon is moved through different interaction sites in the microcomposite until it either escapes the system or its weight diminishes below 10^−4^ times its initial weight at the time of launching. If the photon exits the system, diffuse transmittance or reflectance, depending on the exiting direction, is incremented by the residual weight. This allows for a simultaneous computation of reflectance, transmittance and absorbance throughout a multilayer system although for our purpose we consider here only a single layer of microcomposite.

We note here that the original Monte Carlo model developed by Wang *et al*.^39^ is modified in our study to correct for the specular reflectance from the first layer that is assumed to be non-absorbing in their model. See Supplementary Information (SI) for details on the modification and the validation of the modifed Monte Carlo model through a comparison with results for the optical spectra of composites obtained using the four-flux method for titanium dioxide and vanadium dioxide nanoparticle inclusions (SI Figure 1)^14, 37^.

The effective input parameters for the microcomposite layer required for use in the Monte Carlo model are calculated using the Maxwell-Garnett effective medium theory (MG-EMT)^62^ and the Mie scattering theory^53^. This is accomplished by following the steps outlined in the flowchart shown in Fig. 1b. Scattering and absorption coefficients per unit length *μ*_sca_ and *μ*_abs_ for the spherical semiconductor microparticles are calculated as

3 $${\mu }_{\mathrm{sca}/\mathrm{abs}}=\frac{3}{2}\frac{f{Q}_{\mathrm{sca}/\mathrm{abs}}}{d},$$

where *f* is the volume fraction of the particle inclusions, *d* their diameter, and, *Q*_sca_ and *Q*_abs_ are their scattering and absorption efficiencies respectively.

### Mie scattering

The Mie efficiencies *Q*_sca_ and *Q*_abs_, in turn, are calculated by solving the wave equation for the electric and magnetic fields of a plane electromagnetic wave incident on a spherical scatterer. The spherical scatterer itself and the surrounding medium are assumed to be linear, isotropic and homogeneous wherein the wave equations for the **E** and **H** fields are given by

4 $${\nabla }^{2}{\bf{E}}+{{\rm{k}}}^{2}{\bf{E}}=\mathrm{0,}\quad {\nabla }^{2}{\bf{H}}+{{\rm{k}}}^{2}{\bf{H}}=0$$

Here, *k* ^2^ = *ω* ^2^ *ε*_m_ *μ*_m_ with *ω* as the frequency of the incident electromagnetic wave, and, *ε*_m_ and *μ*_m_ as the permittivity and the permeability of the medium respectively. The time harmonic electric and magnetic fields (**E**,**H**) associated with the incident electromagnetic wave are divergence free in the absence of free charges or currents and related to each other through

5 $$\nabla \times {\bf{E}}=i\omega {\mu }_{{\rm{m}}}{\bf{H}},\quad \nabla \times {\bf{H}}=-i\omega {\varepsilon }_{{\rm{m}}}{\bf{E}}\mathrm{.}$$

The wave equation for the case of scattering from a sphere can be solved using variable separation in spherical coordinates and an expansion of the incident plane wave in vector spherical harmonics. Here, we only present a very brief outline of the cumbersome procedure employed for obtaining a solution while referring the reader to the text by Bohren and Huffman^53^ for more details. The solutions to the wave equations (4) in terms of the vector spherical harmonics **M** and **N** are obtained through the use of the odd (*ψ*_*omn*_) and even (*ψ*_*emn*_) scalar generating functions given by

6 $${\psi }_{emn}=\,\cos (m\varphi ){P}_{n}^{m}(\cos \,\theta ){z}_{n}(kr),\quad {\psi }_{omn}=\,\sin (m\varphi ){P}_{n}^{m}(\cos \,\theta ){z}_{n}(kr).$$

Here, *z*_*n*_(*kr*) is either a spherical Bessel or a Hankel function, and, *m* and *n* are the separation constants for the variables *θ* and *r* of the spherical coordinate system. The vector spherical harmonics **M** and **N** are related to the generating functions through

7 $${{\bf{M}}}_{emn}=\nabla \times ({\bf{r}}{\psi }_{emn}),\quad {{\bf{M}}}_{omn}=\nabla \times ({\bf{r}}{\psi }_{omn})$$

8 $${{\bf{N}}}_{emn}=\frac{\nabla \times {{\bf{M}}}_{emn}}{k},\quad {{\bf{N}}}_{omn}=\frac{\nabla \times {{\bf{M}}}_{omn}}{k}$$

The internal (**E**_int_, **H**_int_) and the scattered fields (**E**_sc_, **H**_sc_) are obtained using the fields (**E**_inc_, **H**_inc_) of the incident electromagnetic radiation upon an application of the boundary conditions

9 $$({{\bf{E}}}_{{\rm{inc}}}+{{\bf{E}}}_{{\rm{sc}}}-{{\bf{E}}}_{{\rm{int}}})\times {\hat{{\bf{e}}}}_{r}=({{\bf{H}}}_{{\rm{inc}}}+{{\bf{H}}}_{{\rm{sc}}}-{{\bf{H}}}_{{\rm{int}}})\times {\hat{{\bf{e}}}}_{r}=0$$

at the interface between the sphere and the surrounding medium. The expansion of the scattered fields (**E**_sc_, **H**_sc_) using the boundary conditions (Equations 9) in terms of the orthogonal vector spherical harmonics gives rise to non-zero coefficients for *m* = 1 alone and can be written out as

10 $${{\bf{E}}}_{{\rm{sc}}}=\sum _{n=1}^{\infty }{E}_{n}(i{a}_{n}{{\bf{N}}}_{e1n}-{b}_{n}{{\bf{M}}}_{o1n})$$

11 $${{\bf{H}}}_{{\rm{sc}}}=\frac{k}{\omega {\mu }_{{\rm{m}}}}\sum _{n=1}^{\infty }{E}_{n}(i{b}_{n}{{\bf{N}}}_{o1n}+{a}_{n}{{\bf{M}}}_{e1n}\mathrm{).}$$

Here, *E*_*n*_ = *i* ^*n*^ *E*_*o*_(2*n* + 1)/[*n*(*n* + 1)] and *E*_*o*_ is the amplitude of the electric field of the incident electromagnetic wave. The vector spherical harmonics (**N**, **M**) derive their radial dependence from the Hankel functions *h*_*n*_(*kr*) = *j*_*n*_(*kr*) + *iy*_*n*_(*kr*) of order *n* wherein *j*_*n*_ and *y*_*n*_ are the spherical Bessel functions of the first and second kind respectively. The Mie scattering coefficients *a*_*n*_ and *b*_*n*_ are then obtained by substituting the expressions for the incident (**E**_inc_, **H**_inc_), internal (**E**_int_, **H**_int_) and scattered fields (**E**_sc_, **H**_sc_) in equations 9 and resolving them into linear equations corresponding to the different components along the unit vectors of the spherical coordinate system. This gives

12 $${a}_{n}=\frac{{\mu }_{m}{n}_{{\rm{r}}}^{2}{j}_{n}({n}_{{\rm{r}}}x)[x{j}_{n}(x)]^{\prime} -{\mu }_{{\rm{s}}}\,{j}_{n}(x)[{n}_{{\rm{r}}}x{j}_{n}({n}_{{\rm{r}}}x)]^{\prime} }{{\mu }_{m}{n}_{{\rm{r}}}^{2}{j}_{n}({n}_{{\rm{r}}}x)[x{h}_{n}(x)]^{\prime} -{\mu }_{{\rm{s}}}{h}_{n}(x)[{n}_{{\rm{r}}}x{j}_{n}({n}_{{\rm{r}}}x)]^{\prime} }$$

13 $${b}_{n}=\frac{{\mu }_{{\rm{s}}}\,{j}_{n}({n}_{{\rm{r}}}x)[x{j}_{n}(x)]^{\prime} -{\mu }_{m}\,{j}_{n}(x)[{n}_{{\rm{r}}}x{j}_{n}({n}_{{\rm{r}}}x)]^{\prime} }{{\mu }_{{\rm{s}}}\,{j}_{n}({n}_{{\rm{r}}}x)[x{h}_{n}(x)]^{\prime} -{\mu }_{m}{h}_{n}(x)[{n}_{{\rm{r}}}x{j}_{n}({n}_{{\rm{r}}}x)]^{\prime} }\mathrm{.}$$

Here, *n*_r_ = *n*_s_/*n*_m_ is the relative refractive index, *n*_s_( = *η*_s_ + *iκ*_s_) is the complex refractive index of the semiconductor microinclusion, *μ*_s_ is the permeability of the spherical particle respectively, and, the primes indicate differentiation of the argument in the square parentheses with respect to the size parameter *x* of the particle. For our simulations, however, we assume both the host medium and the semiconductor microinclusions to be non-magnetic *i*.*e*. *μ*_*m*_ = *μ*_s_ = 1. The scattering (*Q*_sca_) and absorption (*Q*_abs_) efficiencies can thus be computed from the Mie coefficients *a*_*n*_ and *b*_*n*_ (Equations 12–13) for the electric and magnetic fields respectively using

14 $${Q}_{{\rm{sca}}}=\frac{2}{{x}^{2}}\sum _{n}\mathrm{(2}n+\mathrm{1)(}{|{a}_{n}|}^{2}+{|{b}_{n}|}^{2}),$$

15 $${Q}_{{\rm{abs}}}=\frac{2}{{x}^{2}}\sum _{n}\mathrm{(2}n+\mathrm{1)(}{\rm{Re}}({a}_{n}+{b}_{n})-({|{a}_{n}|}^{2}+{|{b}_{n}|}^{2}\mathrm{)).}$$

The order *n* represents the various modes of the plasmonic resonance such as dipole (*n* = 1), quadrupole (*n* = 2), octupole (*n* = 3), and so on.

The conditions for the scattering resonances to occur require that either the denominators of Equations 12 and 13 vanish or achieve a minima. This gives the following two conditions corresponding to the Mie coefficients *a*_*n*_ and *b*_*n*_ respectively for resonant scattering to occur for the nonmagnetic particle and host medium

16 $$\frac{[x{h}_{n}(x)]^{\prime} }{{h}_{n}(x)}=\frac{[{n}_{{\rm{r}}}x{j}_{n}({n}_{{\rm{r}}}x)]^{\prime} }{{n}_{{\rm{r}}}^{2}{j}_{n}({n}_{{\rm{r}}}x)}$$

17 $$\frac{[x{h}_{n}(x)]^{\prime} }{{h}_{n}(x)}=\frac{[{n}_{{\rm{r}}}x{j}_{n}({n}_{{\rm{r}}}x)]^{\prime} }{{j}_{n}({n}_{{\rm{r}}}x)}$$

Further, the scattering anisotropy factor *g* in terms of the Mie coefficients is given by

18 $$g=\frac{4}{{x}^{2}{Q}_{{\rm{sca}}}}\sum _{n}[\frac{n(n+\mathrm{2)}}{n+1}{\rm{Re}}({a}_{n}{a}_{n+1}^{\ast }+{b}_{n}{b}_{n+1}^{\ast })+\frac{2n+1}{n(n+\mathrm{1)}}{\rm{Re}}({a}_{n}{b}_{n}^{\ast })]\mathrm{.}$$

An alternative measure of the scattering anisotropy are the forward ﻿(*Q*_fs_)﻿ and back ﻿(*Q*_bs_) scattering efficiencies given by^63^

19 $${Q}_{{\rm{f}}{\rm{s}}}=\frac{1}{{x}^{2}}|\sum _{n}(2n+1)[{a}_{n}+{b}_{n}]{|}^{2}$$

20 $${Q}_{{\rm{b}}{\rm{s}}}=\frac{1}{{x}^{2}}|\sum _{n}(2n+1){(-1)}^{n}[{a}_{n}-{b}_{n}]{|}^{2}.$$

### Effective refractive index

The real part of the effective refractive index for the microcomposites is calculated from the MG-EMT formula by using the dielectric permittivities of the bulk materials comprising the host and the semiconducting microinclusions. MG-EMT approximates inhomogeneous materials as homogeneous media with effective macroscopic dielectric permittivities. The effective permittivity *ε*_MG_ for a host material with spherical inclusions according to the MG formula is^62^

21 $${\varepsilon }_{{\rm{MG}}}={\varepsilon }_{{\rm{h}}}+3f{\varepsilon }_{{\rm{h}}}\frac{{\varepsilon }_{{\rm{s}}}-{\varepsilon }_{{\rm{h}}}}{{\varepsilon }_{{\rm{s}}}+2{\varepsilon }_{{\rm{h}}}-f({\varepsilon }_{{\rm{s}}}-{\varepsilon }_{{\rm{h}}})},$$

where *f* is the volume fraction of the particulate inclusions and *ε*_s_ is the complex permittivity of the semiconductor microinclusions. In our simulations, the semiconducting spherical microinclusions are the sole contributors to the scattering and absorption of the incident thermal radiation in the composite layer as the host medium is non-absorbing and non-scattering. Therefore, *Q*_sca_ and *Q*_abs_ obtained from the Mie theory using an algorithm by Wiscombe^64^, describe the scattering and absorption in the entire medium.

The Maxwell-Garnett formula is based on the dipolar response of non-interacting particles to an applied electromagnetic field and its use therefore must be limited to small volume fractions (*f* ≤ 0.1) of particle inclusions. It is also well-established that classical EMTs ignore size-dependent properties of particle inclusions leaving them exclusively applicable to weakly scattering systems with particles of radii much smaller than the wavelength *λ* of the incident radiation (*r* < 0.1*λ*)^14^. Thus, here we use the absorption coefficients calculated using the absorption efficiencies *Q*_abs_ (Equation 3) from the Mie theory to account for the size-dependent properties of the microinclusions in the composites in both the Monte Carlo model and the Fresnel equations. Furthermore, to understand and isolate the effect of enhanced scattering from the semicondutor microinclusions, we compare our results obtained from the Monte Carlo modeling with those computed using the Fresnel equations^65^ that account for interference effects alone.

### Reflectance efficiency

We also define a thermal reflectance efficiency factor *η* to quantify and evaluate the suitability of a given low-bandgap semiconductor material for use as microparticle inclusions in composites for thermal insulation. The efficiency factor *η* describes the fraction of the incident radiation being reflected over the entire spectrum and is defined as

22 $$\eta =\frac{{\int }_{{\lambda }_{0}}^{{\lambda }_{1}}R(\lambda )I({T}_{s},\lambda )d\lambda }{{\int }_{{\lambda }_{0}}^{{\lambda }_{1}}I({T}_{s},\lambda )d\lambda }.$$

where *R*(*λ*) is the reflectance obtained from a microcomposite for a given wavelength *λ*. The irradiance *I*(T_*s*_, *λ*), calculated using Planck’s law, corresponds to the spectral density of the electromagnetic radiation emitted by a black body source at temperature *T*_s_.

## Electronic supplementary material

Supplementary Information
