# Supplementary material for: Plasmonically Enhanced Reflectance of Heat Radiation from Low-Bandgap Semiconductor Microinclusions
Source: Sci Rep. 2017 Jul 18;7:5696. doi: 10.1038/s41598-017-05630-4 (PMC5515976; doi:10.1038/s41598-017-05630-4)
Supplement: Supplementary file 1 — Supplementary Information [file 41598_2017_5630_MOESM1_ESM.pdf]

Supplementary Information: Plasmonically Enhanced Reflectance of Heat Radiation from Low-Bandgap Semiconductor Microinclusions

Janika Tang[1,*], Vaibhav Thakore[1,*], and Tapio Ala-Nissila[1,2,3]

[1]COMP CoE at the Department of Applied Physics, Aalto University School of Science, FIN-00076 Aalto, Espoo, Finland

[2]Department of Physics, Brown University, Providence, Rhode Island 02912-1843, USA

[3]Department of Mathematical Sciences and Department of Physics, Loughborough University, Loughborough LE11 3TU, UK

[*]vthakore@knights.ucf.edu and janika.tang@aalto.fi

July 6, 2017

Modification of the Monte Carlo method for modeling thermal radiation transport

The Monte Carlo method developed by Wang et al. [1] for modeling radiation transport in multilayered turbid media is modified and adapted to compute spectral transmittance, reflectance and absorbance for a free-standing layer of insulating dielectric composite with low-bandgap semiconducting microinclusions. The method by Wang et al. [1] for the computation of the specular reflectance considers the first layer of the multilayer system to be non-absorbing. However, this is not valid for the case of a freely suspended absorbing composite layer considered in our work and thus the specular reflectance R_{sp} is modified to

$$R_{\mathrm{sp}} = \frac{(n_0 - \eta_{\mathrm{L}})^2 + \kappa_{\mathrm{L}}^2}{(n_0 + \eta_{\mathrm{L}})^2 + \kappa_{\mathrm{L}}^2}, \tag{1}$$

where η_{L} and κ_{L} are the real and imaginary parts of the refractive index of the composite layer and n_0 is the refractive index of the non-absorbing ambient medium.

To validate the modified Monte Carlo model, we performed simulations for dielectric composites with titanium dioxide (TiO_2) and vanadium dioxide (VO_2) nanoparticle inclusions of various sizes. The corresponding results were then compared against the results obtained using the Fresnel equations and the four-flux method by Laaksonen et al. [2]. The optical parameters for TiO_2 and the semiconducting and metallic phases of thermochromic VO_2 were obtained from the references [3,4] respectively. The nanoparticle inclusions were assumed to be embedded in a host medium of refractive index $n_{\mathrm{m}} = 1.5$. The volume fraction of the nanoinclusions used in the simulations and the composite layer thickness were specified as $f = 0.01$ and $t = 10$ μm respectively. A grid resolution of $dz = 0.1$ μm and $dr = 5$ μm was used for the radial $\hat{z}$ and axial $\hat{r}$ directions respectively (main text, see Figure 1a). The total number of grid elements in the $\hat{r}$-direction was set to $N_r = 100$ while the number of grid elements N_z in the $\hat{z}$-direction was determined by the thickness of the microcomposite layer. The angular dependence of the reflectance and transmittance spectra on the photon-exiting direction $\hat{\alpha}$ was ignored. Each simulation was carried out using 10^7 photons. Similar to the computation of the optical parameters by Laaksonen *et al.* [2], the scattering μ_{sca} and absorption μ_{abs} coefficients, and, the scattering anisotropy g were computed using Mie theory for use with the Monte Carlo method (main text, see Figure 1b). As in Laaksonen *et al.* [2], unlike the study presented here, the optical parameters employed in the Fresnel equations were computed using equations (3) and (7) in the main text based on the MG-EMT for validation.

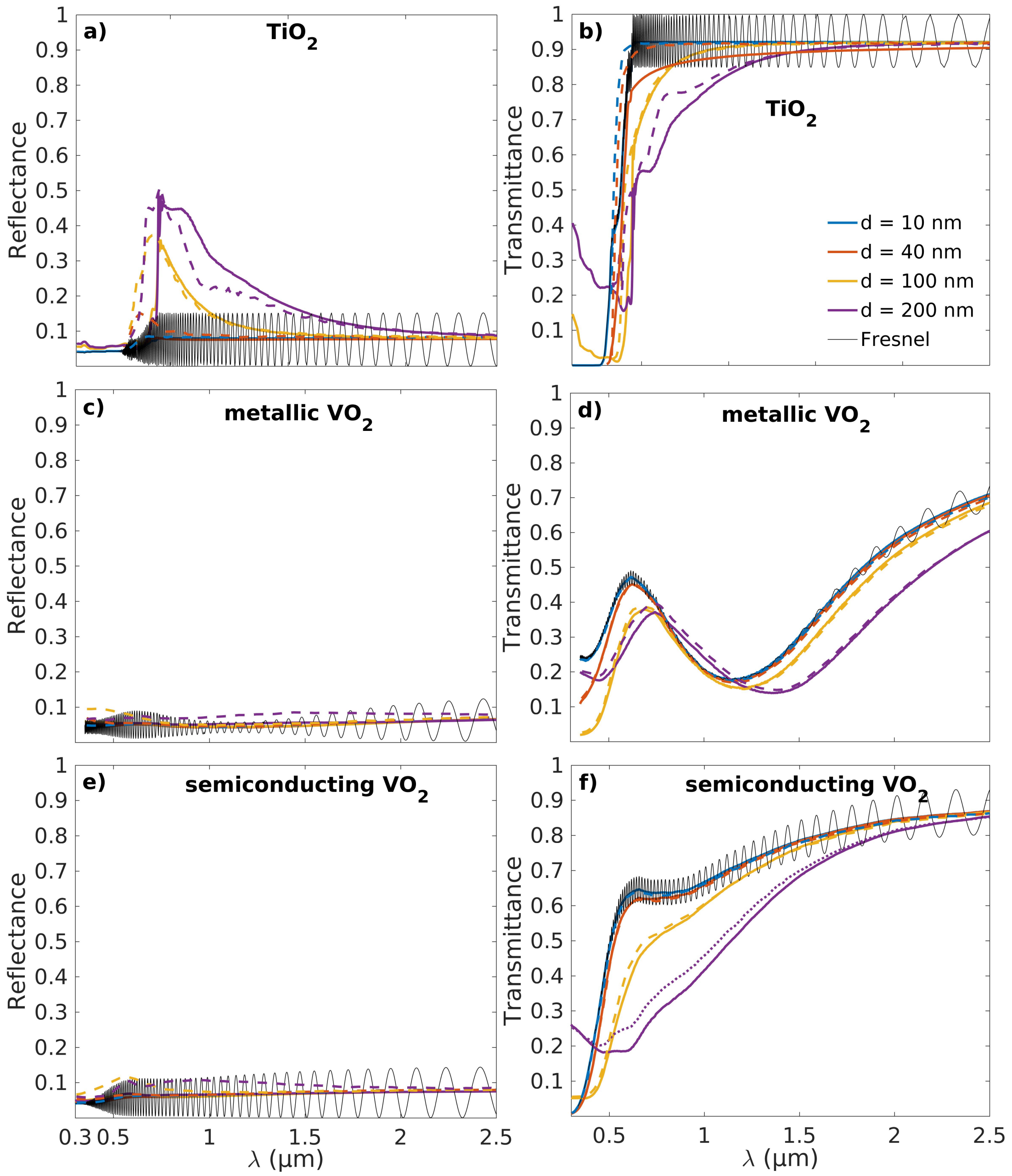

Figure 1: **Validation of the modified Monte Carlo model.** Spectral transmittance and reflectance for **(a-b)** TiO_2, **(c-d)** metallic VO_2, and **(e-f)** semiconducting VO_2 nanoparticles with volume fraction $f = 0.01$ embedded in a dielectric host medium of refractive index $n = 1.5$ and thickness $t = 10$ μm calculated using the Monte Carlo method (solid lines), the four-flux method (dashed lines) and the Fresnel equations (black lines).

SI Figure 1a-f shows comparisons of the transmittance and reflectance spectra for the TiO_2 and VO_2 nanocomposites computed using the Fresnel equations [5], the modified Monte Carlo and the four-flux methods [2]. A comparison between the computed spectra from the four-flux and the Monte Carlo methods shows that the agreement with the results from the Fresnel equations is better for the Monte Carlo model at smaller wavelengths in the regime of low scattering (SI Figure 1). Both the four-flux and the Monte Carlo methods capture the average spectral behavior quite accurately for the smaller nanoinclusions of size $d = 10\,$nm at longer wavelengths wherein interference effects dominate the optical spectra obtained from the Fresnel equations. It must be noted that unlike the Fresnel equations both the four-flux and the Monte Carlo models of radiative transfer do not account for the interference effects. The deviations from the results obtained using Fresnel equations for the four-flux and the Monte Carlo methods become

more significant due to increased scattering effects with an increase in the size of the nanoinclusions. There are also some differences between the results from the four-flux and the Monte Carlo methods. These differences in transmittance and reflectance spectra are more pronounced in the case of composites with TiO_2 nanoinclusions (SI Figure 1a-b) that exhibit enhanced scattering while the agreement between the two methods is much better for the metallic and semiconducting forms of the thermochromic VO_2 nanocomposites characterized by lower scattering (SI Figure 1c-f). This difference in optical spectra obtained from the four-flux and the Monte Carlo methods is observed because of the assumptions made in the four-flux method with regard to the value of the average path-length parameter and the ratio of the forward scattering radiation to that of the collimated light [2]. The Monte Carlo method, on the other hand, does not make any such assumptions and is known to be more accurate than the four-flux-method [6].

Optical parameters of semiconductor materials used for microinclusions in the insulating composites

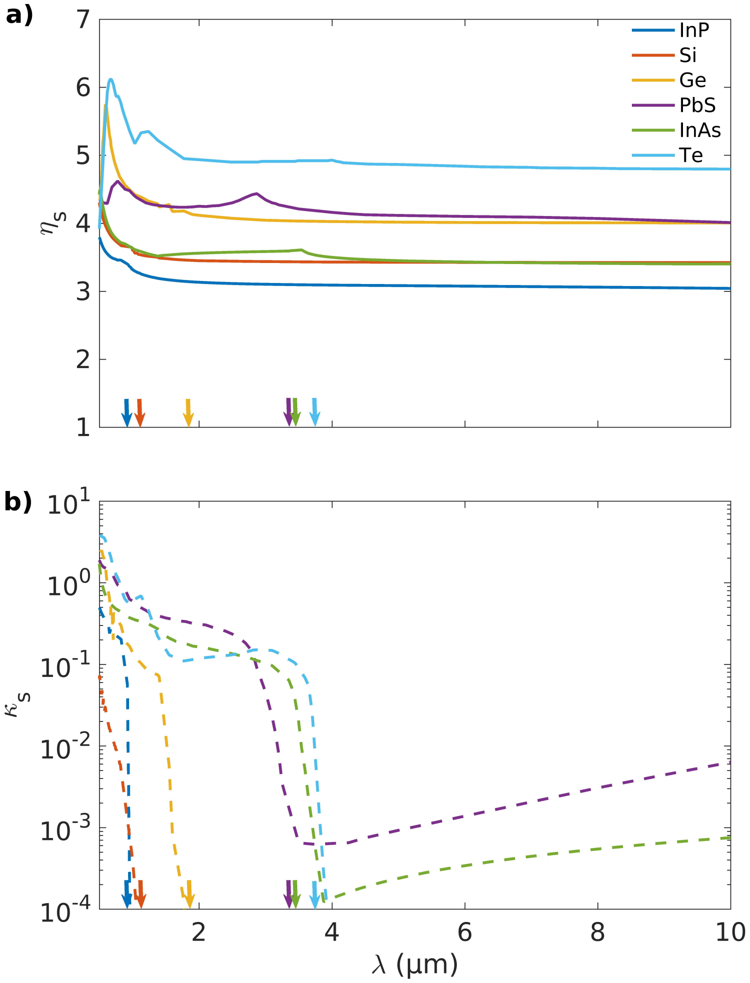

Figure 2: **Bulk optical parameters of low bandgap semiconductor materials used for microinclusions in insulating composites.** **(a)** Real part of the refractive index η_s, and, **(b)** Imaginary part of the refractive index κ_s as a function of the wavelength λ of the incident thermal radiation. Close to the absorption band edge (marked by the vertical colored arrows) a steep increase in both the real (η_s) and imaginary (κ_s) parts of the refractive indices is observed for all low band gap semiconductor materials due to an increase in the number of free charge carriers. Additionally, PbS and InAs exhibit a continuous linear increase in κ_s beyond the absorption band edge at longer wavelengths, likely due to defect states within the bandgap.

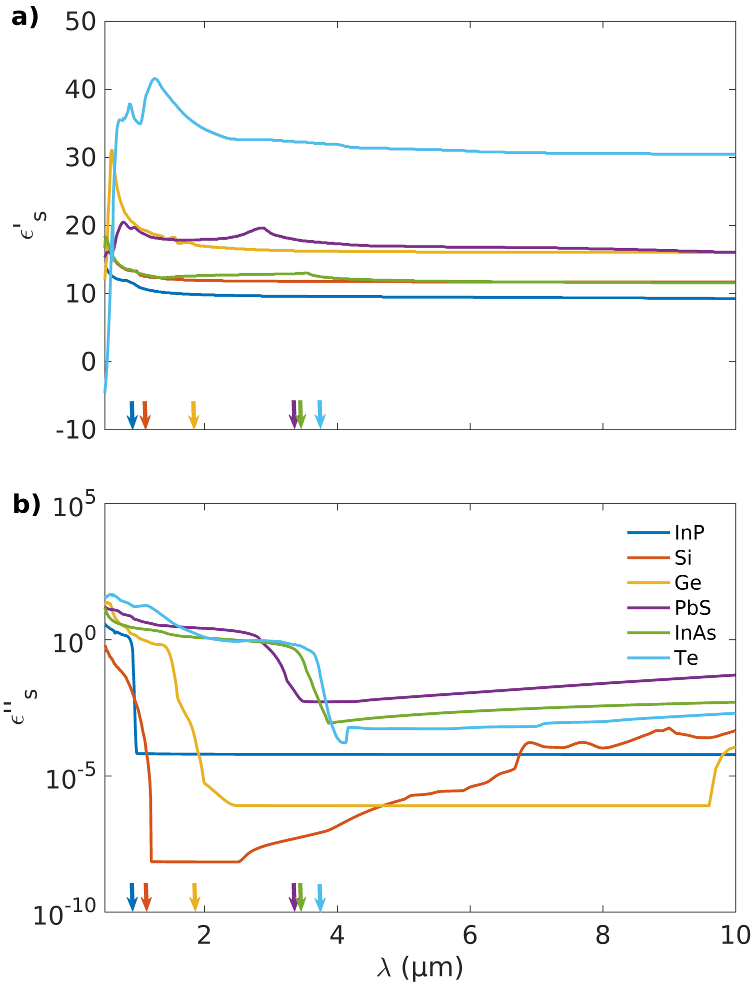

Figure 3: **Bulk values of the dielectric permittivity ϵ_s of low bandgap semiconductor materials used for microinclusions in insulating composites.** **(a)** Real part of the permittivity $\epsilon_{s'}$, and, **(b)** Imaginary part of the permittivity $\epsilon_{s''}$ for InP, Si, Ge, PbS, InAs and Te as a function of the wavelength λ of the incident thermal radiation.

Mie scattering from semiconductor microinclusions

SI Figure 5 and Figure 2e in the main text, show that forward scattering dominates over backscattering. Furthermore, a deconvolution of the back scattering efficiency in to contributions from the dipole, quadrupole and octupole modes shows that the octupole modes occur close to the bandgap wavelengths ($\lambda \approx \lambda_{\text{bg}}$), are more sharply peaked and also the strongest followed by quadrupole and dipole modes that occur at longer wavelengths. SI Table 1 shows that the strongest radiative damping for all Mie modes occurs for Si microinclusions followed by other materials. A strong correlation is also seen between the reflectance efficiency η (main text, Figure 8) and the strength of the radiative damping relative to dissipation ϵ''_s in a semiconductor microinclusion (SI Table 1).

Table 1: A comparison of the results for the radiative damping ($x_n^{2n+1}/n[(2n-1)!!]^2$) for the strongest of the dipole, quadrupole and the octupole modes (main text, Figure 2e and SI Figure 4) with the dissipation in the semiconductor microinclusions ($\epsilon_s'' = 2\eta_s\kappa_s$, SI Figure 3b).

Dipole modes ($n = 1$)				
Material	λ_n (μm)	x_n	$\frac{x_n^{2n+1}}{n[(2n-1)!!]^2}$	$\epsilon_s''(\lambda_n)$
InP	1.77	1.60	4.08	$6.30 \cdot 10^{-5}$
Si	5.46	1.45	3.05	$2.40 \cdot 10^{-6}$
Ge	2.53	1.19	1.69	$8.14 \cdot 10^{-7}$
PbS	5.35	1.18	1.64	$8.77 \cdot 10^{-3}$
InAs	8.95	1.47	3.20	$4.38 \cdot 10^{-3}$
Te	7.05	0.83	0.57	$7.11 \cdot 10^{-4}$

Quadrupole modes ($n = 2$)				
Material	λ_n (μm)	x_n	$\frac{x_n^{2n+1}}{n[(2n-1)!!]^2}$	$\epsilon_s''(\lambda_n)$
InP	1.35	2.09	2.24	$6.40 \cdot 10^{-5}$
Si	4.11	1.93	1.47	$1.99 \cdot 10^{-7}$
Ge	1.92	1.57	0.53	$5.57 \cdot 10^{-5}$
PbS	4.05	1.56	0.51	$5.32 \cdot 10^{-3}$
InAs	6.85	1.93	1.47	$2.92 \cdot 10^{-3}$
Te	5.0	1.17	0.12	$5.40 \cdot 10^{-4}$

Octupole modes ($n = 3$)				
Material	λ_n (μm)	x_n	$\frac{x_n^{2n+1}}{n[(2n-1)!!]^2}$	$\epsilon_s''(\lambda_n)$
InP	1.11	2.55	1.03	$6.52 \cdot 10^{-5}$
Si	3.28	2.41	0.71	$4.51 \cdot 10^{-8}$
Ge	1.54	1.96	0.16	$6.47 \cdot 10^{-2}$
PbS	3.22	1.96	0.17	$4.87 \cdot 10^{-2}$
InAs	5.50	2.40	0.68	$1.99 \cdot 10^{-3}$
Te	3.89	1.50	0.03	$1.27 \cdot 10^{-3}$

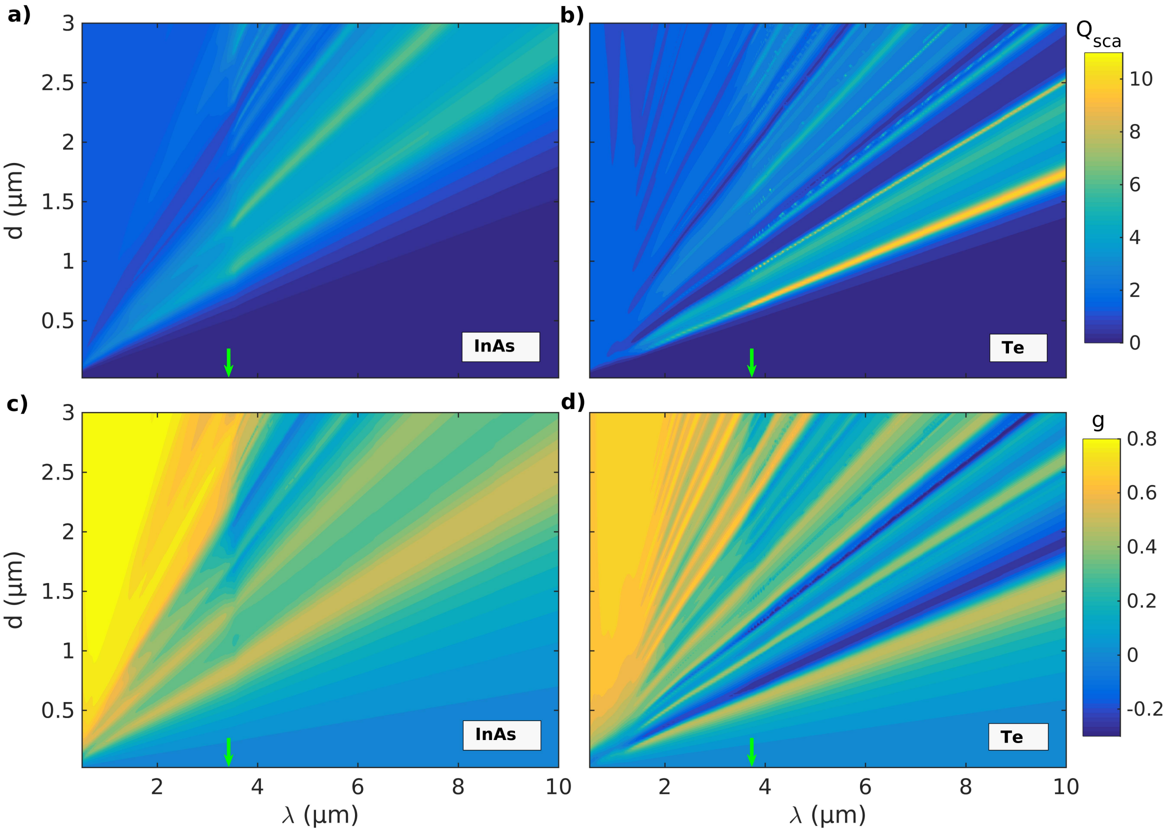

Figure 4: **Effect of the change in the wavelength of the incident radiation and the microinclusion diameter on the Mie scattering parameters.** **(a-b)** Scattering efficiency Q_{sca}, and, **(c-d)** anisotropy factor g as a function of the wavelength λ of the incident thermal radiation and the diameter d of spherical InAs and Te microinclusions, respectively. Similar to the plots of Q_{sca} and g for InP, Si, Ge and PbS microinclusions (main text, Figure 4), the bandgap wavelengths λ_{bg} (indicated by vertical green arrows) for the semiconductor materials here denote a transition from low to high Q_{sca} and strongly forward ($+g$) to mixed scattering regimes for the microinclusions with increasing λ.

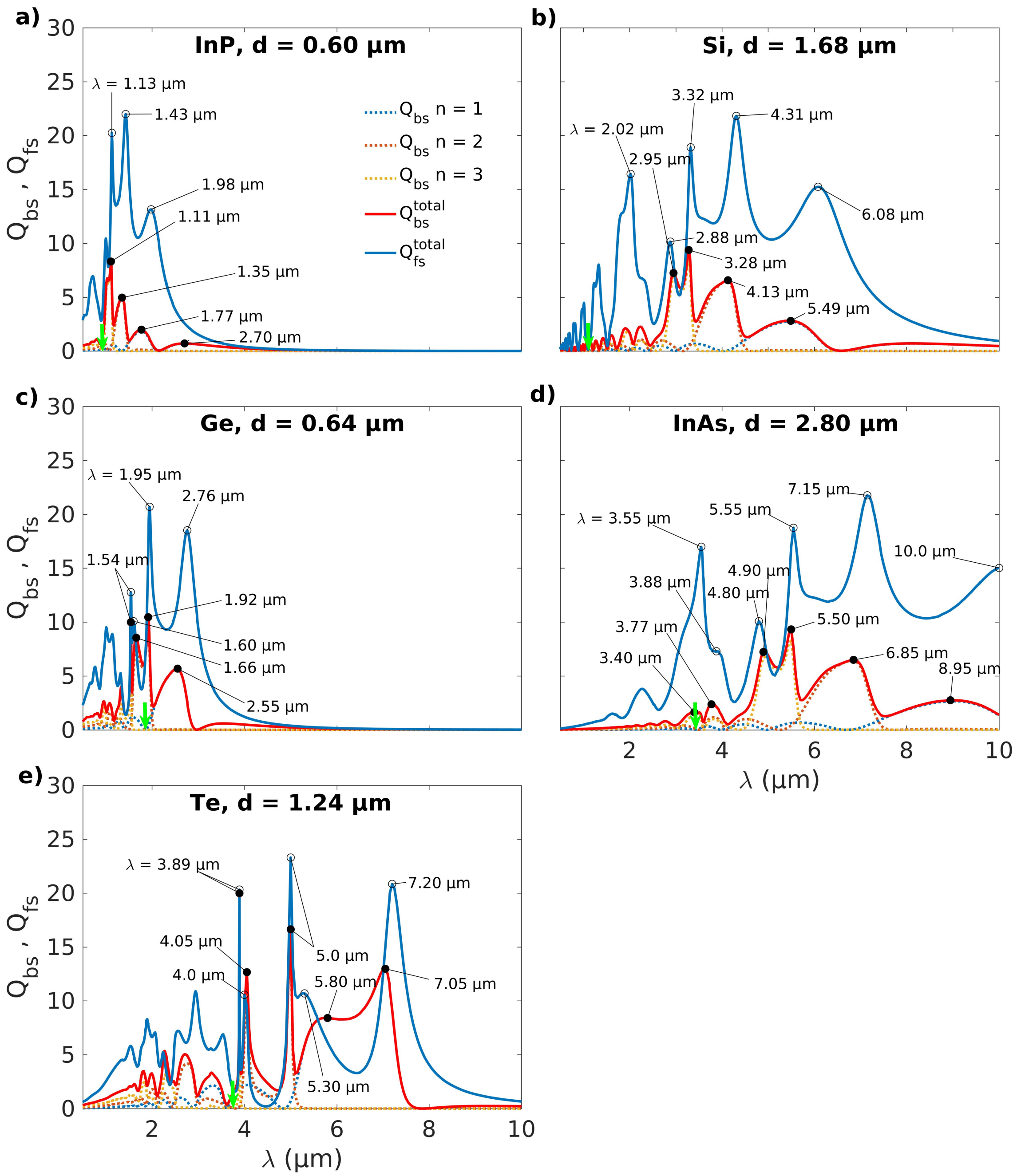

Figure 5: **Effect of the change in the wavelength of the incident thermal radiation on the forward (Q_{fs}) and back (Q_{bs}) scattering parameters for various semiconductor microinclusions.** Forward and back scattering efficiencies along with a deconvolution of Q_{bs} into contributions from the dipole and higher order modes ($n = 1, 2,$ and 3) as a function of the wavelength λ for semiconductor microinclusions of **(a)** InP, **(b)** Si, **(c)** Ge, **(d)** InAs and **(e)** Te with diameter d corresponding to the minima ($g_{\min}$) in the scattering anisotropy. The dotted blue, red and yellow curves represent contributions from the dipole ($n = 1$), quadrupole ($n = 2$) and octupole ($n = 3$) modes to the back scattering efficiency Q_{bs}, respectively. The vertical green arrows represent the bandgap wavelengths λ_{bg} for the low bandgap semiconductors.

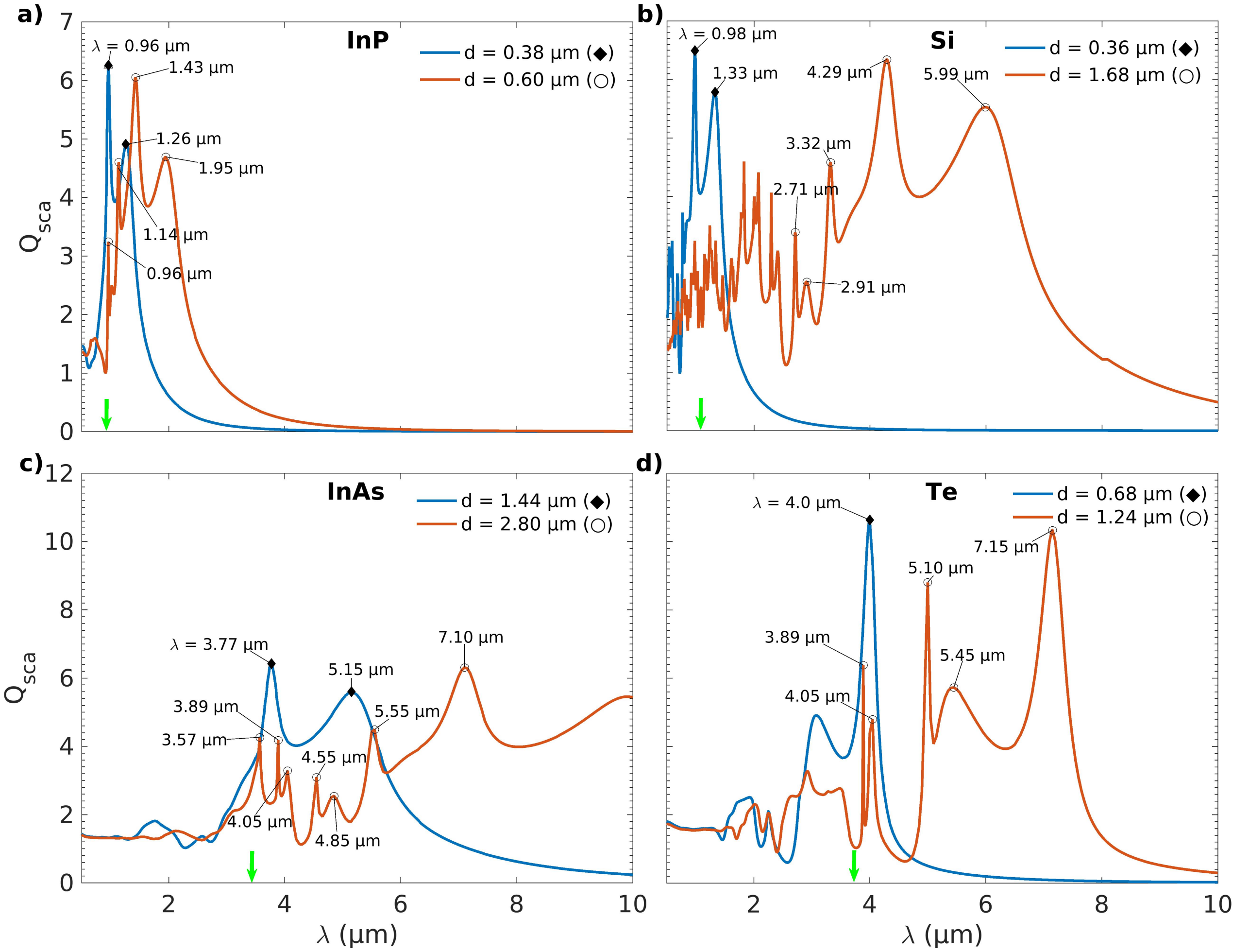

Figure 6: **Effect of the semiconductor microinclusion size on the Mie scattering efficiency.** Mie scattering efficiencies Q_{sca} for **(a)** InP, **(b)** Si, **(c)** InAs, and, **(d)** Te microinclusions of sizes d corresponding to $Q_{\mathrm{sca}}^{\mathrm{max}}$ and g_{min}, respectively (main text, Figure 3 and Tables 1 and 2). The vertical green arrows indicate the bandgap wavelengths λ_{bg}. Sharp Fano resonances in Q_{sca} are observed for the composites with Si, InAs and Te microinclusions with an increase in the particle size.

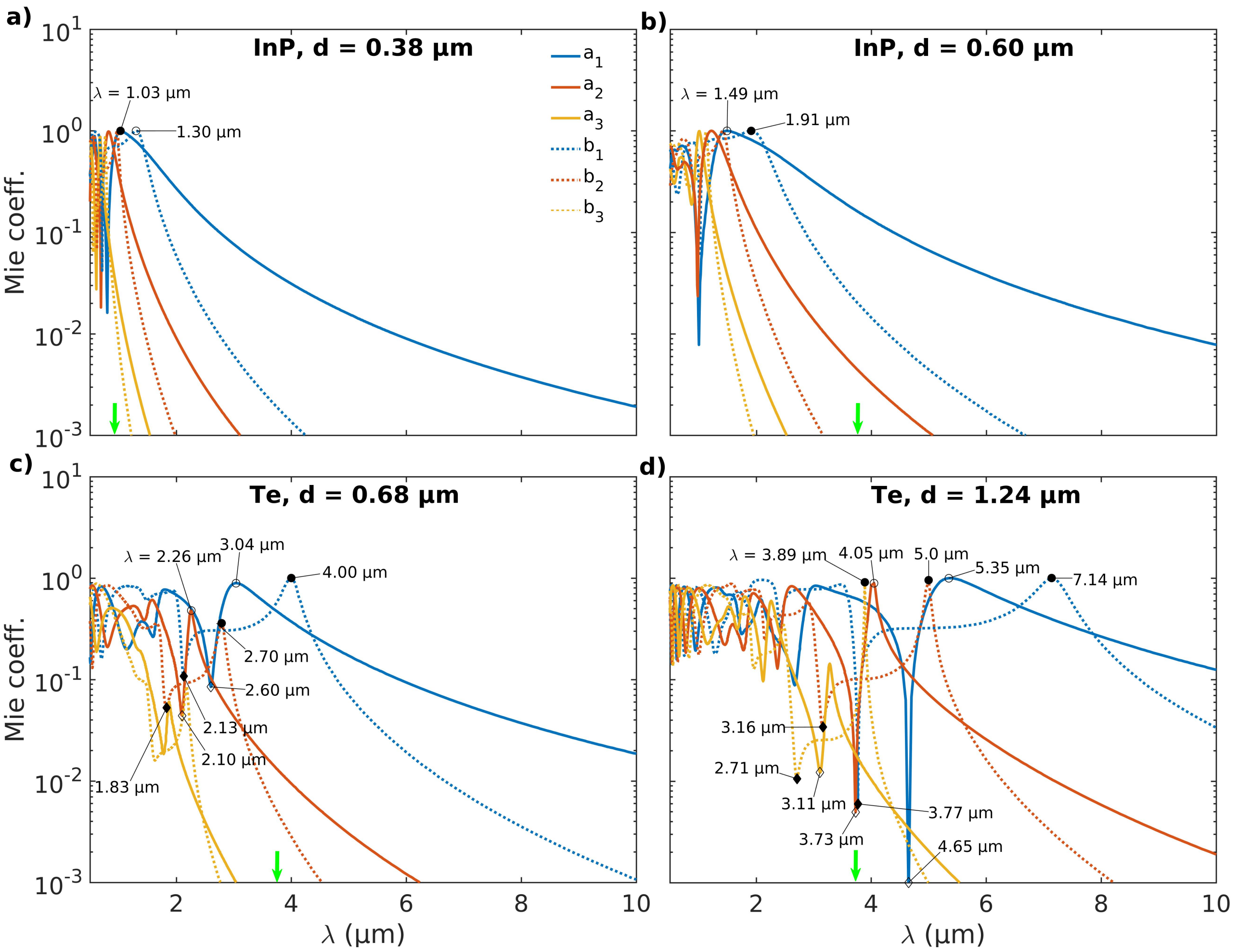

Figure 7: **Effect of the semiconductor microinclusion size on the electric and magnetic Mie coefficients.** Mie coefficients a_n (solid-line) and b_n (dashed-line) as a function of wavelength λ for spherical microinclusions of **(a, b)** InP, and, **(c, d)** Te for particle diameters d corresponding to $Q_{\text{sca}}^{\text{max}}$ and g_{min}, respectively (main text Figure 2 and Tables 1 and 2). The vertical green arrows indicate the bandgap wavelengths λ_{bg}. Similar to the results reported for Si, Ge, PbS and InAs microinclusions (main text, Figure 5), an increase in the microinclusion size d is accompanied by a strengthening of the magnetic modes b_n.

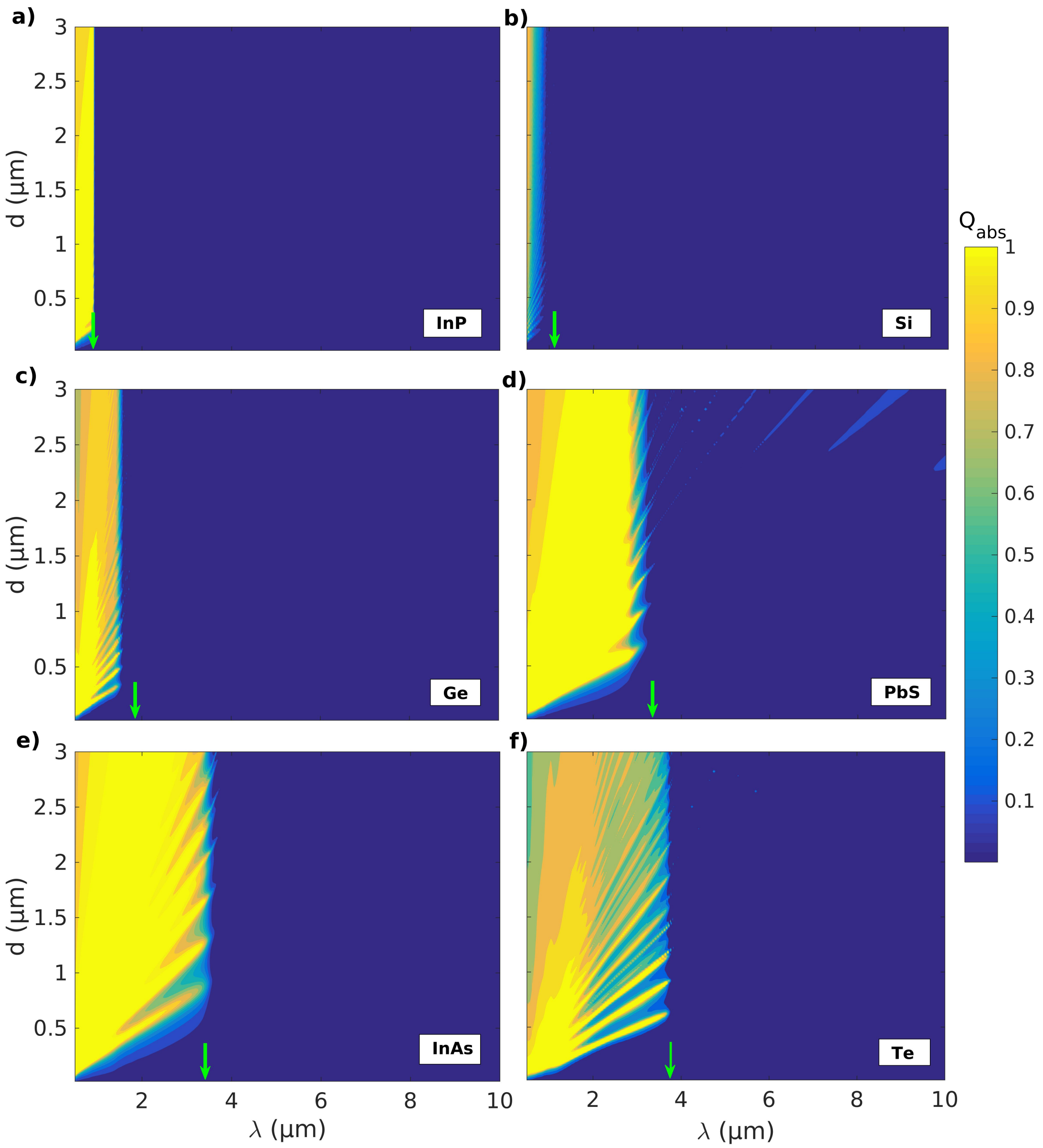

Figure 8: **Effect of the change in the wavelength of the incident radiation and the semiconductor microinclusion diameter on the Mie absorption efficiency.** Absorption efficiency Q_{abs} as a function of wavelength λ and diameter d of spherical **(a)** InP, **(b)** Si, **(c)** Ge, **(d)** PbS, **(e)** InAs, and, **(f)** Te microinclusions. The vertical green arrows on the x-axis indicate the bandgap wavelengths λ_{bg} marking the onset of the absorption edge.

SI Figures 4a-d show the scattering efficiency Q_{sca} and the anisotropy factor g for Te and InAs microinclusions of diameters varying between $d = 0.02$ to 3 μm in the wavelength range $\lambda = 0.5$ to 10 μm for the incident thermal radiation. The scattering anisotropy g is observed to be strongly forward-scattering after the absorption edge for sizes of microinclusions that are comparable to the wavelength of the incident thermal radiation (SI Figure 4c-d). Similar to the PbS microinclusions (main text, Figures 3d, and, 4b, d), free charge carriers created in the conduction or valence bands at $\lambda \gtrsim \lambda_{\mathrm{bg}}$ (SI Figure 8e-f) and due to weak absorption peaks (SI Figure 9c-d) outside of the main absorption band at longer wavelengths result in plasmonic resonances that give rise to large peaks in Q_{sca} for Te and InAs microinclusions (SI Figures 4a-b and 6c-d, respectively). As pointed out in the main text, the features in Q_{sca} and g, such as maxima and minima, are seen to redshift and broaden to varying degrees with an increase in

the size d of the microinclusions for all materials studied here (main text, Figure 3 and SI Figures 4 and 6). Also, it is clearly seen that $Q_{\rm sca}$ in SI Figure 4a-b obtains lower values for the wavelengths $\lambda < \lambda_{\rm bg}$ for both InAs and Te. The reduction in $Q_{\rm sca}$ for InAs and Te microinclusions follows an increase in the absorption as seen in SI Figure 8e-f.

SI Figure 7 presents the Mie coefficients of modes $n \leq 3$ for InP and Te microinclusions of size $d_{Q_{\rm sca}^{\rm max}}$ and $d_{g_{\rm min}}$ that correspond to the highest and the lowest values of the scattering efficiency $Q_{\rm sca}^{\rm max}$ and the anisotropy factor g_{min} respectively (Tables 1 and 2, main text). It can be seen that the sharp peaks in the magnetic and electric modes give rise to peaks in scattering efficiencies $Q_{\rm sca}$. For example, the peaks in Mie coefficients at $\lambda = 7.14, 5.35, 5.0, 4.05$ and 3.89 μm (SI Figure 7b) for Te microinclusions of size $d = 1.24$ μm ($\circ$) contribute to resonances in $Q_{\rm sca}$ at $7.15, 5.45, 5.0, 4.05$ and 3.89 μm (SI Figure 6d) respectively.

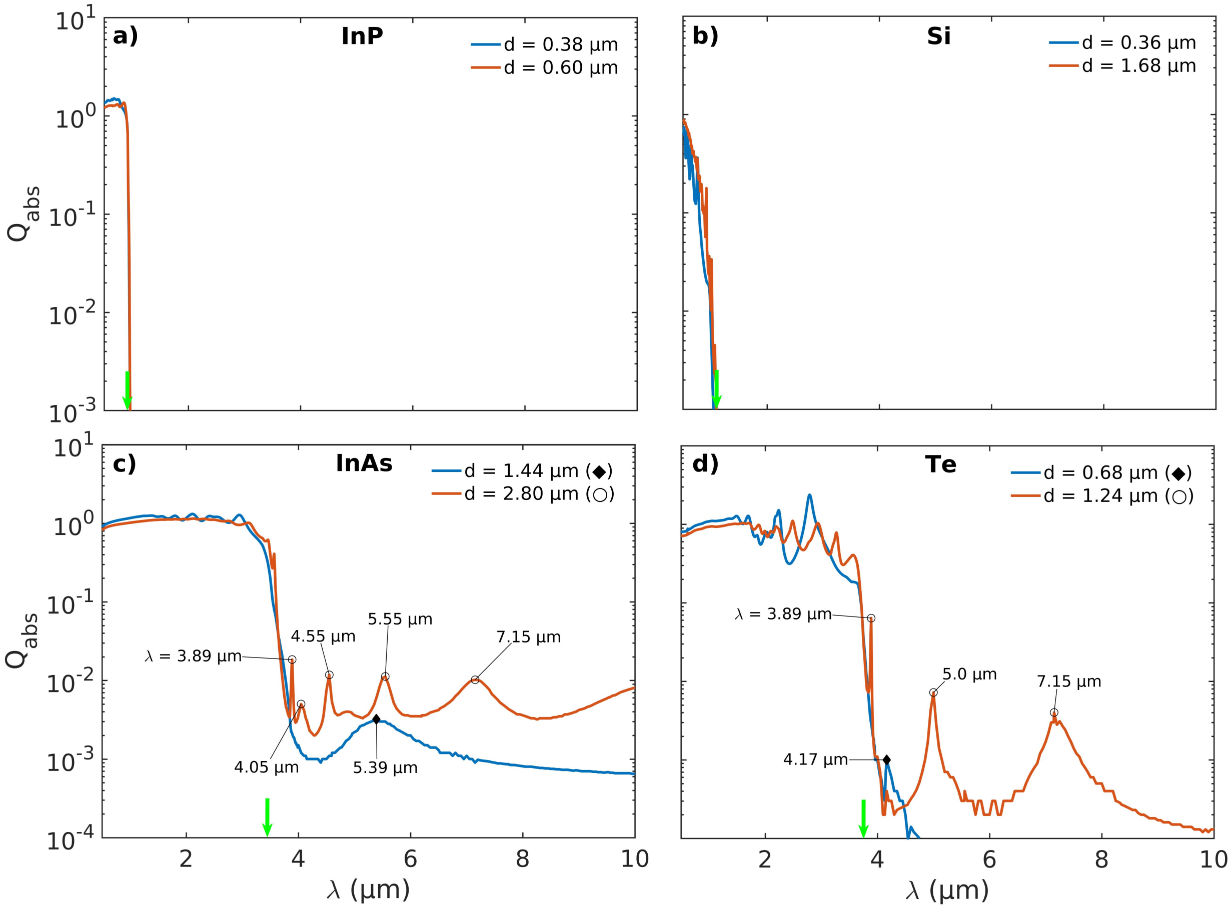

Figure 9: **Effect of the particle size on the Mie absorption efficiency for different semiconductor microinclusions.** Absorption efficiency $Q_{\rm abs}$ for various sizes of **(a)** InP, **(b)** Si, **(c)** InAs and **(d)** Te microinclusions of sizes d corresponding to $Q_{\rm sca}^{\rm max}$ and $g_{\rm min}$, respectively (main text, Figure 2 and Tables 1 and 2). The vertical green arrows indicate the bandgap wavelengths $\lambda_{\rm bg}$. Contributions to $Q_{\rm abs}$ due to weak absorption from the defect states in composites with InAs and Te microinclusions serve to significantly broadband the reflectance of the incident thermal radiation.

Spectral reflectance of microcomposites

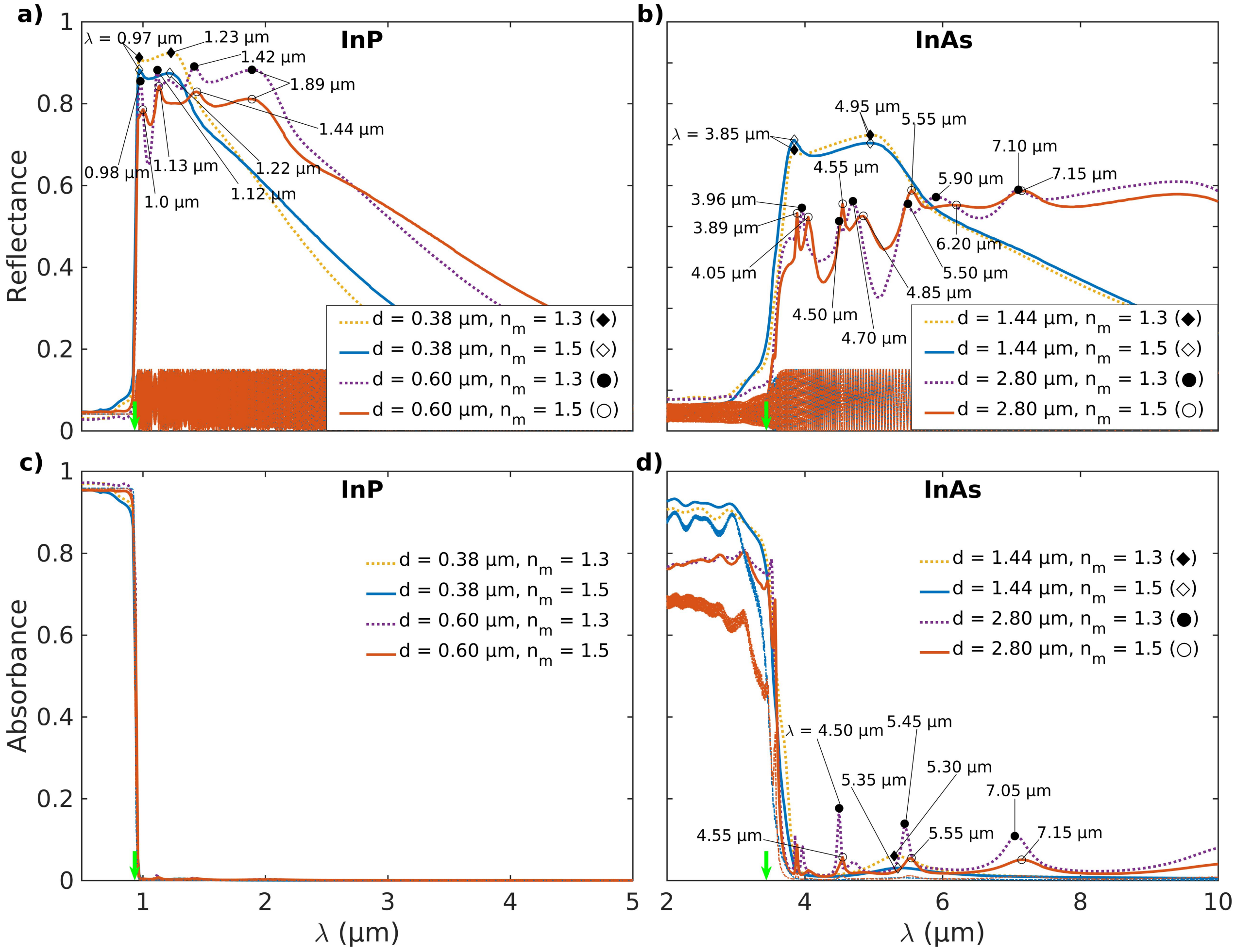

Figure 10: **Spectral behavior of insulating composites with low-bandgap semiconductor microinclusions and the effect of an increase in the refractive index of the host medium.** The spectral reflectance and absorbance of microcomposites with **(a, c)** InP, and **(b, d)** InAs spherical inclusions of diameter d and volume fraction $f = 0.01$ embedded in a dielectric host medium of refractive indices $n_m = 1.5$ and 1.3 and thickness $t = 200$ μm. The solid lines correspond to results from Monte Carlo simulations for the case of host refractive index $n_m = 1.5$ while the thin dotted lines of the same color denote results from Fresnel equations. The vertical green arrows on the x-axis indicate the bandgap wavelengths λ_{bg}. The broadbanding of the reflectance peaks in microcomposites with InAs inclusions correlates well with the weak peaks in absorbance that occur away from the main absorption band at longer wavelengths.

Scattering and absorption from the low-bandgap semiconducting microinclusions significantly increases reflectance and absorbance as can be seen from a comparison of the spectra obtained using the Monte Carlo method and the Fresnel equations in SI Figure 10. Again, the difference between the two methods emphasizes the huge impact a small volume fraction of particle inclusions has on the infrared spectra of the microcomposites with semiconductor microinclusions. High and broad maxima in reflectance spectra are observed for both InP and InAs microcomposites especially for the larger microinclusions (SI Figure 10a-b).

For composites with InAs microinclusions, similar to composites with PbS particles (main text, Figure 6b), the peaks in reflectance (SI Figure 10b) that occur beyond the absorption band edge at longer wavelengths ($n_m = 1.5$ ($\circ$, $\diamond$); $\lambda = 7.15, 5.55, 4.85$ and 4.55 μm for $d = 2.80$ μm ($\circ$); and, $\lambda = 4.95$ and 3.85 μm for $d = 1.44$ μm ($\diamond$)) correlate well with the resonances in Q_{sca} ($\lambda = 7.10, 5.55, 4, 85$ and 4.55 μm for $d = 2.80$ μm($\circ$), and, $\lambda = 5.15$ and 3.77 μm for $d = 1.44$ μm ($\blacklozenge$), SI Figure 6c). These scattering resonances are observed to arise due to enhanced

scattering from the excitation of plasmonic resonances (○, •) that are identified in the plots for Mie coefficients (main text, Figure 5g-h and SI Figure 7c-d). On the other hand, similar to composites with Ge microparticles (main text, Figure 5c-d), the InP microinclusions do not exhibit any plasmonic resonances in the Mie coefficients a_n (○) and b_n (•) away from the main absorption band (SI Figure 7a-b). This behavior is consistent with the absence of absorption away from the main absorption band in InP microinclusions (SI Figures 9a and 10c). Thus, the reflectance spectra for microcomposites with Ge (main, text Figure 6a) and InP inclusions (SI Figure 10a) is marked by an absence of the broadbanding that is observed for composites with PbS (main text, Figure 6b), InAs (SI Figure 10b) and Te microinclusions (main text, Figure 7a). However, the peaks in reflectance ($n_m = 1.5$ (○, ◇); $\lambda = 1.89, 1.44, 1.13$ and 1.0 μm for $d = 0.60$ μm (○), and, $\lambda = 1.22$ and 0.97 μm for $d = 0.38$ μm (◇), SI Figure 10a) that occur for microcomposites with InP inclusions correlate well with peaks in Q_{sca} ($\lambda = 1.95, 1.43, 1.14$ and 0.96 μm for $d = 0.60$ μm (○), and $\lambda = 1.26$ and 0.96 μm for $d = 0.38$ μm (♦), SI Figure 6a) that arise from the resonances (○, •) observed in the Mie coefficients (SI Figure 7a-b). Again, similar to other microcomposites, a redshifting and broadening of reflectance peaks is also observed for composites with InP and InAs microinclusions with an increase in the particle size d (SI Figure 10a-b).

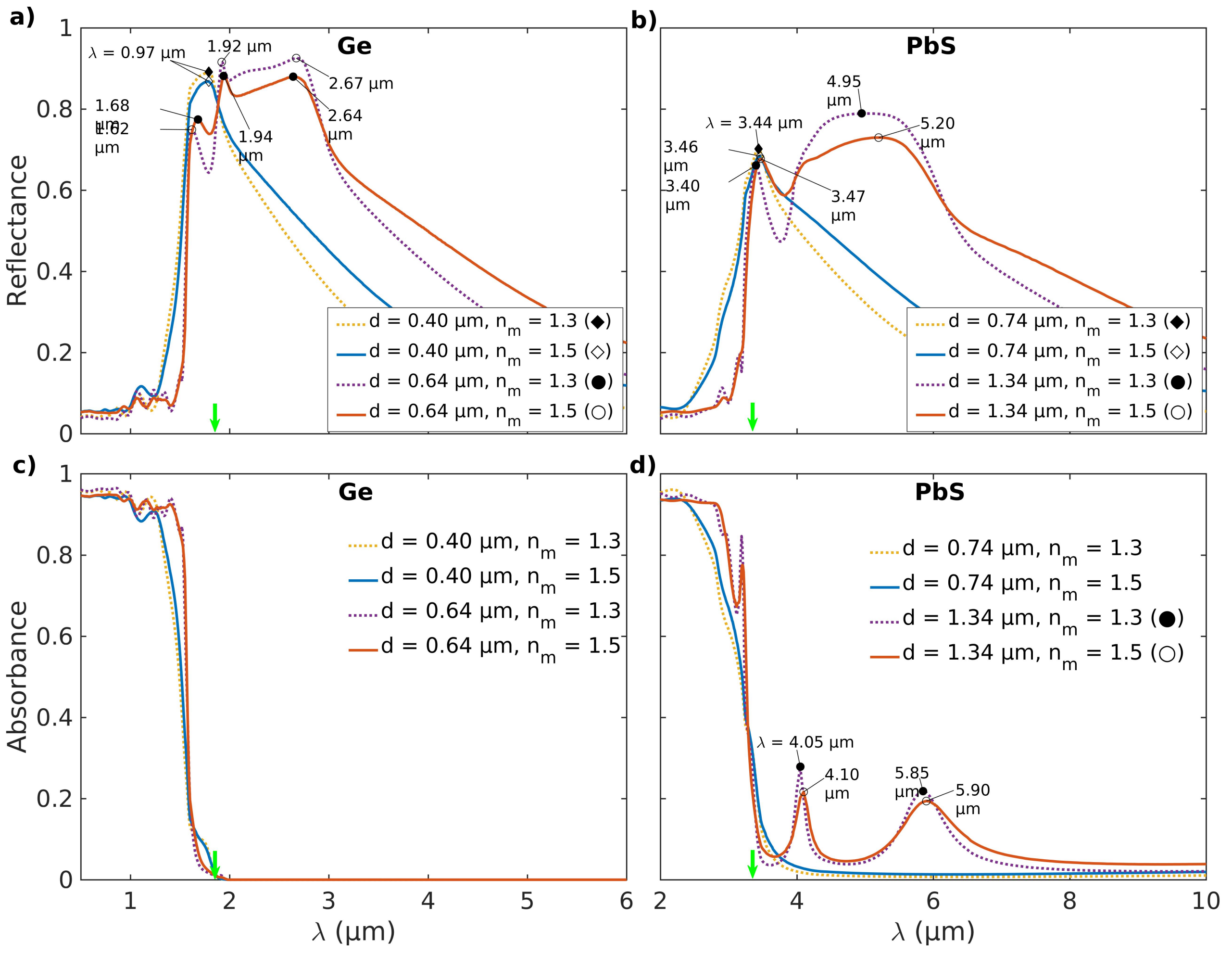

Figure 11: **Effect of an increase in the refractive index of the host medium on the optical spectra of the microcomposites due to plasmonic resonances.** The spectral reflectance and absorbance of microcomposites with **(a, c)** Ge and **(b, d)** PbS spherical inclusions of diameter d and volume fraction $f = 0.01$ embedded in dielectric media of refractive indices $n_m = 1.5$ (solid-lines) and 1.3 (dotted-lines). The vertical green arrows on the x-axis indicate the bandgap wavelengths λ_{bg}. A red-shift in the reflectance peaks denotes the surface-localized nature of plasmonic resonances.

Nature of plasmonic resonances

SI Figures 10a-b and 11 show a comparison of the reflectance spectra for composites with InP, InAs and Ge, PbS microinclusions with an increase in the refractive index of the host medium from $n_m = 1.3$ (•, ⧫) to 1.5 (○, ◇). Results show that for the smaller microinclusions there occurs no discernible redshift in the reflectance peaks with an increase in the host-medium refractive index. For example, the position of the reflectance peaks at $\lambda = 0.97$ and 1.23 μm remains unchanged for microcomposites with InP inclusions of size $d = 0.38$ μm (⧫, ◇) with an increase in the host refractive index (Figure 10a). Similarly, in the case of composites with InAs particles of size $d = 1.44$ μm (⧫, ◇) the reflectance peaks at $\lambda = 3.85$ and 4.95 μm do not exhibit any redshift (Figure 10b). However, for the larger microinclusions (•, ○) a distinct red shift of the reflectance peaks is seen in all microcomposites except perhaps for the broadest of peaks that occur at longer wavelengths (main text, Figure 7a-b and SI Figures 10a-b, 11a-b). Again taking microcomposites with InP particles of size $d = 0.60$ μm (•, ○) as an example it is observed that the reflectance peaks at $\lambda = 1.42, 1.12$ and 0.98 μm redshift to $\lambda = 1.44, 1.13$, and 1.0 μm (Figure 10a). For the broadest reflectance peak at $\lambda \approx 1.9$ μm, however, it is difficult to make out the redshift (Figure 10a). Thus, these results point to a transformation in the nature of plasmonic resonances from volume modes to localized surface modes driven by a strengthening of the magnetic Mie modes (main text, Figure 5 and SI Figure 4) as pointed out in the main text.

References

[1] L. H. Wang, S. L. Jacques, L. Q. Zheng, *Computer Methods and Programs in Biomedicine* **1995**, *47*, 131–146.

[2] K. Laaksonen, S. Y. Li, S. R. Puisto, N. K. J. Rostedt, T. Ala-Nissila, C. G. Granqvist, R. M. Nieminen, G. A. Niklasson, *Sol. Energy Mater. Sol. Cells* **2014**, *130*, 132–137.

[3] E. D. Palik, *Handbook of Optical Constants of Solids*, Academic Press, Orlando, **1985**.

[4] N. R. Mlyuka, G. A. Niklasson, C. G. Granqvist, *physica status solidi (A)* **2009**, *206*, 2155–2160.

[5] O. S. Heavens, *Optical properties of thin solid films*, Dover Publications, 1965th ed., **1991**.

[6] B. Maheu, J. P. Briton, G. Gouesbet, *Appl. Opt.* **1989**, *28*, 22–24.
